# Thermally activated delayed phosphorescence triggered by charge separation state carrier storage in an organic scintillator

**DOI:** 10.1093/nsr/nwaf045

**Published:** 2025-02-12

**Authors:** Ruo-Yu Cao, Yu-Bing Si, Qi Yang, Zi-Ying Gao, Jia-Wang Yuan, Yi Zhao, Qiu-Chen Peng, Kai Li, Shuang-Quan Zang, Ben Zhong Tang

**Affiliations:** Tianjian Laboratory of Advanced Biomedical Sciences, Henan Key Laboratory of Crystalline Molecular Functional Materials, College of Chemistry, Zhengzhou University, Zhengzhou 450001, China; Tianjian Laboratory of Advanced Biomedical Sciences, Henan Key Laboratory of Crystalline Molecular Functional Materials, College of Chemistry, Zhengzhou University, Zhengzhou 450001, China; Tianjian Laboratory of Advanced Biomedical Sciences, Henan Key Laboratory of Crystalline Molecular Functional Materials, College of Chemistry, Zhengzhou University, Zhengzhou 450001, China; Tianjian Laboratory of Advanced Biomedical Sciences, Henan Key Laboratory of Crystalline Molecular Functional Materials, College of Chemistry, Zhengzhou University, Zhengzhou 450001, China; Tianjian Laboratory of Advanced Biomedical Sciences, Henan Key Laboratory of Crystalline Molecular Functional Materials, College of Chemistry, Zhengzhou University, Zhengzhou 450001, China; State Key Laboratory of Physical Chemistry of Solid Surfaces, iChEM, Fujian Provincial Key Lab of Theoretical and Computational Chemistry, and College of Chemistry and Chemical Engineering, Xiamen University, Xiamen 361005, China; Tianjian Laboratory of Advanced Biomedical Sciences, Henan Key Laboratory of Crystalline Molecular Functional Materials, College of Chemistry, Zhengzhou University, Zhengzhou 450001, China; Tianjian Laboratory of Advanced Biomedical Sciences, Henan Key Laboratory of Crystalline Molecular Functional Materials, College of Chemistry, Zhengzhou University, Zhengzhou 450001, China; Tianjian Laboratory of Advanced Biomedical Sciences, Henan Key Laboratory of Crystalline Molecular Functional Materials, College of Chemistry, Zhengzhou University, Zhengzhou 450001, China; School of Science and Engineering, Shenzhen Institute of Aggregate Science and Technology, The Chinese University of Hong Kong, Shenzhen (CUHK-Shenzhen), Shenzhen 518172, China

**Keywords:** organic scintillators, afterglow, charge separation, thermally activated delayed phosphorescence, X-ray afterglow imaging

## Abstract

Organic scintillators are among the most promising due to their inherent merits in terms of heavy metal–free constituents, synthesis designability, affordability of raw materials, and low usage costs. However, the limited X-ray excited luminescence (XEL) property of organic scintillators affects their application. To date, the main approaches for improving the XEL property of organic scintillators have focused on introducing heavy atoms to increase the absorbance of X-rays and establishing new luminescence pathways, such as thermally activated delayed fluorescence (TADF), to increase the exciton utilization efficiency. Even so, the XEL property of organic scintillators is not ideal compared with that of commercial inorganic scintillators. In this work, a highly stable charge separation (CS) state trap was introduced into the design of an organic scintillator. Combined with a unique thermally activated delayed phosphorescence (TADP) process, highly efficient capture and conversion of high-energy carriers are realized. As a result, the exciton generation efficiency dramatically increases, with an ultrahigh XEL intensity, and X-ray afterglow imaging at room temperature is achieved for the first time. This work provides a brand-new strategy for the design of high-performance organic scintillators.

## Teaser

A highly stable charge separation state trap was introduced into the design of an organic scintillator, resulting in a dramatical increase in X-ray excited luminescence intensity.

## INTRODUCTION

X-ray detection technology has recently received increasing attention in various fields, such as high-energy physics, medical diagnosis, and non-destructive testing [1–5]. As the core materials of X-ray detection technology, scintillators directly determine the detection performance and have become the most important optoelectronic materials in X-ray detection technology [6–11]. Currently, the mainstream research hotspot for scintillators is inorganic semiconductors and inorganic-organic hybrid materials because of their high light yield (LY) and multiple luminescence properties [12–16]. However, the harsh preparation conditions and poor stability of traditional scintillators usually lead to high usage costs. Moreover, the heavy metals contained in these scintillators pose potential environmental pollution risks, greatly limiting their popularity and application. Small-molecule organic materials have many advantages, such as heavy metal–free constituents, synthesis designability, affordable raw materials and low usage costs [17–22]. These materials have achieved great success in various optoelectronic fields, including in organic light-emitting diodes (OLEDs), photovoltaic devices and field-effect transistors, indicating their broad development potential in the field of scintillators.

**Figure 1.**
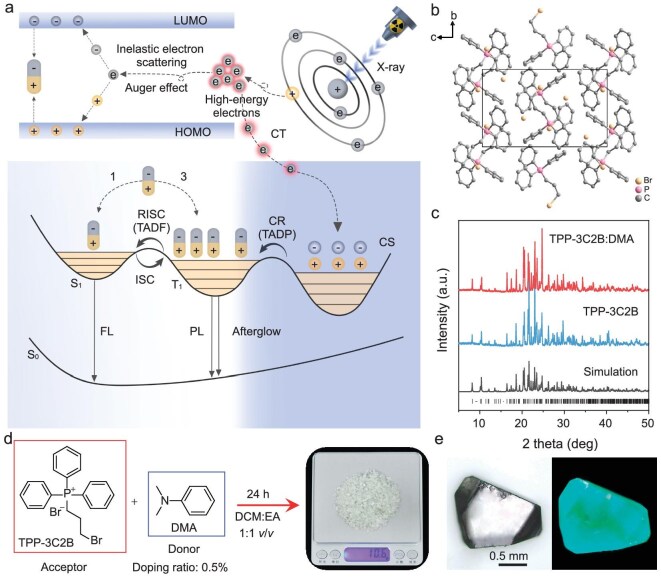
Schematic diagram of the mechanism and molecular structure. (a) Mechanistic diagram of the XEL of TPP-3C2B:DMA. (b) Single-crystal structure of TPP-3C2B:DMA. (c) PXRD data of TPP-3C2B:DMA and TPP-3C2B. (d) Synthesis route of TPP-3C2B:DMA crystals. (e) Photographs of a TPP-3C2B:DMA crystal in bright (left) and dark (right) fields.

The scintillation behaviour of small-molecule organic materials under X-ray irradiation was discovered for anthracene crystals as early as 1947 [23]. However, the development of organic scintillators has been very slow over the past several decades. The X-ray excited luminescence (XEL) mechanism of an organic scintillator is shown in Fig. 1a and can be divided into three stages [24,25]. In the first stage (**Step I**), high-energy X-ray is absorbed by atoms through the photoelectric effect and Compton scattering to produce high-energy electrons. In the second stage (**Step II**), through inelastic electron scattering and the Auger effect, some of the high-energy electrons produce secondary electrons and holes, which interact to produce excitons. In the third stage (**Step III**), visible luminescence is produced by the radiative transition process of the excitons. Owing to the lack of heavy atoms, most organic scintillators exhibit weak X-ray absorption, and the spin-forbidden transition from the triplet excited state to the singlet ground state limits their exciton utilization. In recent years, heavy atoms such as bromine and iodine have been added to organic scintillators to increase their X-ray absorption, whereas fast thermally activated up-conversion processes and aggregation-induced emission moieties have been introduced into the design of organic scintillators to increase exciton utilization [26–29]. However, very few of these materials have conspicuous scintillation properties, and the XEL intensity of most small-molecule organic materials is far lower than that of inorganic materials. The cause of the above results is that in **Step II**, many high-energy electrons with inappropriate energies cannot effectively produce secondary electrons and holes. To solve this problem, a donor-acceptor (D-A)-doped luminescence system of TPP-3C2B:DMA (*N,N*-dimethylaniline) is prepared in this work, in which a charge separation (CS) state trap is constructed to capture high-energy electrons. The unique thermally activated delayed phosphorescence (TADP) process provides a new pathway for significantly enhancing the efficiency of exciton generation, resulting in an ultrahigh XEL intensity. Moreover, the presence of the CS state trap endows the organic scintillator with an ultralong afterglow at room temperature, enabling the realization of X-ray afterglow imaging, which shows potential for application in archaeology, identification of cultural relics and industrial detection.

## RESULTS AND DISCUSSION

### Synthesis and characterization of scintillation properties

**Figure 2.**
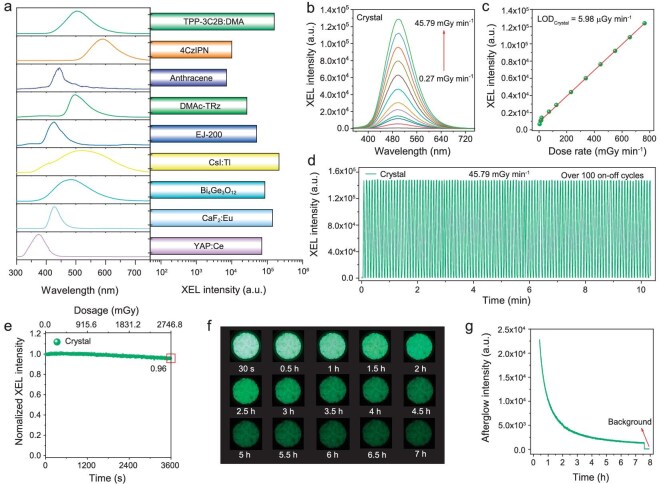
Characterization of the scintillation properties of TPP-3C2B:DMA. (a) Comparison of the scintillation performance of TPP-3C2B:DMA and commercial scintillators at 25°C. (b) XEL spectra of the TPP-3C2B:DMA at different X-ray doses. (c) Dose rate dependence of the XEL intensity of the TPP-3C2B:DMA. (d) XEL intensity of the TPP-3C2B:DMA under X-ray irradiation (45.79 mGy min^−1^) over 100 on-off cycles (each cycle lasted 3 s). (e) XEL intensity of the TPP-3C2B:DMA under continuous X-ray irradiation for 60 min (2746.8 mGy in total). (f) Photos of X-ray excited afterglow of the TPP-3C2B:DMA. (g) Afterglow profile of the TPP-3C2B:DMA after X-ray excitation.

TPP-3C2B:DMA was easily prepared via cocrystallization of TPP-3C2B and DMA [30]. The DMA doping ratio was 0.5%, which could not be determined by single-crystal X-ray diffraction (SCXRD) or powder X-ray diffraction (PXRD) (Fig. 1b and c) but could be confirmed by ^1^H-NMR (Figs S1 and S2 in Supplementary data) and high-performance liquid chromatography (HPLC) (Fig. S3). The method for preparing TPP-3C2B:DMA crystals is very mild and simple, and millimetre-scale crystals at the gram scale can be easily prepared under laboratory conditions using commercial reagents without further purification (Fig. 1d, e and Fig. S4). As shown in Fig. 2a, the XEL intensity of TPP-3C2B:DMA is not only higher than that of the plastic organic scintillator EJ-200 and classical organic scintillators such as anthracene, 4CzIPN and DMAc-TRz but also surpasses that of many commercially available inorganic scintillators such as Bi_4_Ge_3_O_12_, CaF_2_:Eu and YAP:Ce. The LY of TPP-3C2B:DMA is calculated as 65 535 photons MeV^−1^ via a relative method with anthracene, 4CzIPN, DMAc-TRz and CsI:Tl as standard scintillators (Fig. S5) [25]. For medical samples, the X-ray dose should be as low as possible to minimize harm to the human body while ensuring completion of detection. As shown in Fig. 2b, c and Fig. S6, TPP-3C2B:DMA has a sensitive response to X-rays, and its XEL intensity presents a good linear relationship with the X-ray dose. The limit of detection (LOD) of TPP-3C2B:DMA calculated via the 3*σ* method is 5.98 μGy min^−1^, which is far lower than the minimum dose required for medical detection (330 μGy min^−1^) [31]. In addition, for scintillators, stable cycling performance is also a very important usage indicator. As shown in Fig. 2d and e, TPP-3C2B:DMA can maintain its XEL intensity well after being continuously exposed to high-dose X-ray (45.79 mGy min^−1^) for 60 min or after on-off cycling over 100 times. Most importantly, TPP-3C2B:DMA can exhibit a significant afterglow of over 7 h observable by the naked eye and a time curve after the X-ray excitation is stopped (Fig. 2f, g and Video S1), which is the first ultralong afterglow observed for a small-molecule organic material under X-ray excitation.

### Investigation of the scintillation mechanism

To understand the influence of the CS state on the XEL of TPP-3C2B:DMA, undoped TPP-3C2B was selected as a contrasting compound. As shown in Figs S7–S9, the XEL intensity of TPP-3C2B is much lower than that of TPP-3C2B:DMA, although the photoluminescence intensity of the former is still 40% of that of the latter. The X-ray absorbance capacity of TPP-3C2B is almost the same as that of TPP-3C2B:DMA (Fig. S10). Moreover, the electrical conductivities of TPP-3C2B:DMA and TPP-3C2B show analogous increases under X-ray irradiation, suggesting they have similar carrier formation processes (Fig. S11) [32]. Thus, **Step I** is not the origin of their different XEL properties. Moreover, TPP-3C2B and TPP-3C2B:DMA have almost the same crystal structure (Fig. 1b, c, Fig. S12 and Tables S1–S4), ensuring their similar nonradiative transition processes induced by molecular packing. That is, the influence of **Step III** on their different XEL properties can also be excluded. Thus, the difference in XEL properties between TPP-3C2B and TPP-3C2B:DMA is attributed mainly to the ability of the CS state to capture electrons and holes.

**Figure 3.**
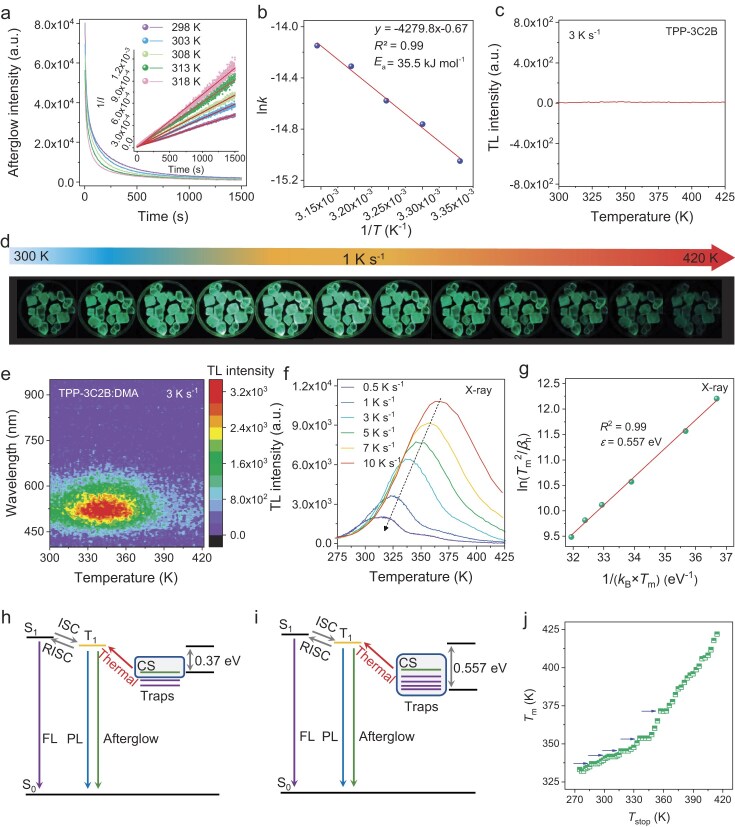
Characterization of the scintillation mechanism of TPP-3C2B:DMA. (a) Time-dependent emission decay curves of TPP-3C2B:DMA at different temperatures after X-ray excitation was stopped. Inset: The curves were fitted with second-order reaction dynamics to calculate *k*. (b) Rate constants of TPP-3C2B:DMA fitted with Arrhenius expressions. (c) TL curve of TPP-3C2B after X-ray excitation. (d) Photos of TPP-3C2B:DMA at temperatures ranging from 300 to 420 K. (e) Intensity-wavelength-temperature TL spectrum of TPP-3C2B:DMA after X-ray excitation. (f) TL curves of TPP-3C2B:DMA after X-ray excitation under different heating rates. (g) Estimation of the trap depth via the Hoogenstraaten method based on the X-ray excited TL data. (h and i) Energy diagram and photophysical processes of TPP-3C2B:DMA after excitation at (h) room temperature and (i) a high temperature. (j) Relationship between *T*_m_ and *T*_stop_. The blue arrows indicate that TPP-3C2B:DMA has at least five traps with different depths from 273 to 425 K.

As shown in Fig. S13, there are two lifetimes in TPP-3C2B, 0.3 μs and 74 ms, corresponding to thermally activated delayed fluorescence (TADF) and phosphorescence, respectively. For TPP-3C2B:DMA, in addition to the two short lifetimes (3.3 μs and 310 ms), an ultralong afterglow is observed (Fig. S14 and Video S2). The origin of the afterglow was investigated via time-dependent emission decay measurements at different temperatures. As shown in Fig. 3a, the afterglow decay curves can be well fit with second-order reaction kinetics (1/*I* ∝ *t*), suggesting that the generation of excitons is a second-order kinetics process. This result is obtained because the recombination process occurs between electrons and holes and because the concentrations of electrons and holes are equal; thus, the reaction rate should be proportional to the square of the electron (or hole) concentration, i.e. a second-order reaction. This result suggests that the CS state can effectively capture and store carriers, which is the origin of the intense XEL.

Furthermore, the activation energy (*E_a_*), i.e. the depth of the CS state trap, can be evaluated according to the Arrhenius expression:


(1) \begin{eqnarray*}
{\rm ln}\ k = \frac{- E_a}{RT} + {\rm constant},
\end{eqnarray*}

where *k* is the rate constant, *T* is the temperature and *R* is the ideal gas constant. The CS state trap depth is calculated to be 35.5 kJ mol^−1^ (0.370 eV) (Fig. 3b). As shown in Fig. S15, the same result can be obtained by fitting the photoluminescence decay curves, demonstrating that the photoexcited afterglow and X-ray excited afterglow have the same source.

The thermoluminescence (TL) curves of TPP-3C2B:DMA were measured to further understand its luminescence process. As shown in Fig. 3c–e, no obvious TL can be found for TPP-3C2B, but significantly TL can be observed for TPP-3C2B:DMA from 300 to 420 K. In the X-ray excited afterglow process, heating or irradiation with near-infrared light can effectively increase the luminescence intensity because these stimuli can accelerate the release of trapped carriers and enhance photon emission (Fig. S16) [33]. A trap depth of 0.557 eV is obtained for TPP-3C2B:DMA via the Hoogenstraaten method (Fig. 3f and g), which is much deeper than the CS state trap (0.370 eV) [34,35]. Similar results are obtained for the analogues of TPP-3C2B:DMA, including TPP-3C2B:DEA and TPP-3C2B:MeDMA (Figs S17–S21 and Table S5), whose trap depths are 0.425 eV and 0.357 eV, respectively, which are both greater than the depths of their CS state traps (0.336 eV and 0.307 eV, respectively). These differences originate from the different photophysical processes that occur under the two different measurements. The trap depth obtained by fitting the afterglow curve is the energy barrier for the transfer of electrons from the CS state to the T_1_ state at approximately room temperature (Fig. 3h). The depth obtained via the Hoogenstraaten method is the energy barrier for the transfer of all electrons in the trap states to the T_1_ state induced by a heating stimulus (Fig. 3i). As shown in Fig. 3j and Fig. S22, the *T*_m_–*T*_stop_ curves indicate that TPP-3C2B:DMA has at least five traps with different depths in the temperature range from 273 to 425 K [36–38], further supporting the proposed mechanism. Similarly, for UV light excited TPP-3C2B:DMA, TL curves were also recorded, and the calculated trap depth is 0.558 eV, which is almost the same as that of X-ray excited TPP-3C2B:DMA (Fig. S23). This result indicates that the same afterglow emits a pathway after X-ray irradiation and UV light irradiation. Moreover, similar results can also be found for the reported D-A doped luminescence system TN@TPBi (Figs S17 and S24).

As shown in Figs S25 and S26, TPP-3C2B and TPP-3C2B:DMA exhibit intense and almost the same XEL at 83 K. With increasing temperature, the XEL of TPP-3C2B rapidly decreases, whereas the XEL of TPP-3C2B:DMA slowly decreases. This phenomenon might originate from a TADP process from the CS state to the first excited triplet state (T_1_). At 293 K, the XEL intensity of TPP-3C2B is only 4% of that at 83 K, whereas the XEL intensity of TPP-3C2B:DMA is 20% of that at 83 K. The similar XEL intensities at 83 K can be attributed to the restriction of intramolecular motion by the low temperature, which blocks the nonradiative transition channel. With increasing temperature, the thermal motion significantly quenches the emission. Moreover, the TADP process leads to an increase in phosphorescence and slows the decrease in XEL. As shown in Fig. S27, the reduction in XEL intensity for TPP-3C2B:DMA with increasing temperature is much slower than that for TPP-3C2B. The same result can be obtained via temperature-dependent photoluminescence measurements (Figs S28–S30). Similar results can also be found for the contrasting compounds (Fig. S31), demonstrating that the TADP induced by the CS state can effectively increase the XEL of organic scintillators.

As shown in Fig. 1a, afterglow production was attributed to a luminescence process including charge transfer (CT), CS and charge recombination (CR). To better understand the luminescence process of TPP-3C2B:DMA, theoretical calculations were carried out. Owing to the presence of Br atoms in TPP-3C2B, the spin-orbit coupling (SOC) between the lowest singlet excited state and the lowest triplet excited state is significant. When the DMA molecule is embedded in a defect of TPP-3C2B, the SOC is barely influenced (Fig. S32a, Tables S6 and S7). As shown in Fig. S32b, the transition from S_*n*_ to T_*n*_ in TPP-3C2B:DMA, as can be inferred from the orbital composition of the excited state, is a typical *n*→π* transition. In this transition, the *n* electrons originate from the *p_x_* and *p_y_* orbitals of the N atom in the DMA molecule and the *p*_x_ and *p*_z_ orbitals of the Br atom in TPP-3C2B. Moreover, the energy levels of S*_n_* and T*_n_* (*n* ≤ 10) simultaneously decrease by ∼0.06 to 0.1 eV when the DMA molecule is doped into TPP-3C2B, which is evidence of weak interactions between DMA and TPP-3C2B, as shown in Table S8 [30,39,40]. Notably, for the dopant-free system TPP-3C2B, the spin density of the triplet state is distributed mainly across TPP-3C2B, and Mulliken charge/spin population analysis reveals an intra-CT state. However, when the electron-donating moiety of DMA is involved, the spin density is prone to localize on the TPP-3C2B and DMA partners, presenting an inter-CT property (Fig. S32c and Tables S9, S10). With these diabatic states established, the energy gap between the first triplet excited state and the CT state is calculated to be 0.324 eV, which is slightly lower than the energy gap between the first triplet excited state and the CS state (0.370 eV). This result suggests that the energy of the CT state of TPP-3C2B:DMA is greater than the energy of its CS state, which fits well with the proposed luminescence mechanism.

In addition, the reported acceptor-doped D-A system TPP:CuI and its analogues BrTPP:CuI and 3BrTPP:CuI were chosen as contrasting compounds, which have highly stable CS state traps and afterglow similar to those of TPP-3C2B:DMA (Fig. S33a and b) [41]. As expected, the XEL intensities of contrasting compounds are significantly greater than those of the undoped systems TPP, BrTPP and 3BrTPP, confirming the universality of our strategy proposed in this work. More interestingly, the magnification of the photoluminescence intensity between the doped system (TPP:CuI, BrTPP:CuI and 3BrTPP:CuI) and the undoped system (TPP, BrTPP and 3BrTPP) is much smaller than that of the XEL intensity, further demonstrating that the stable CS state trap is beneficial for the capture and conversion of high-energy carriers (Fig. S33c–h).

### X-ray imaging and X-ray afterglow imaging

**Figure 4.**
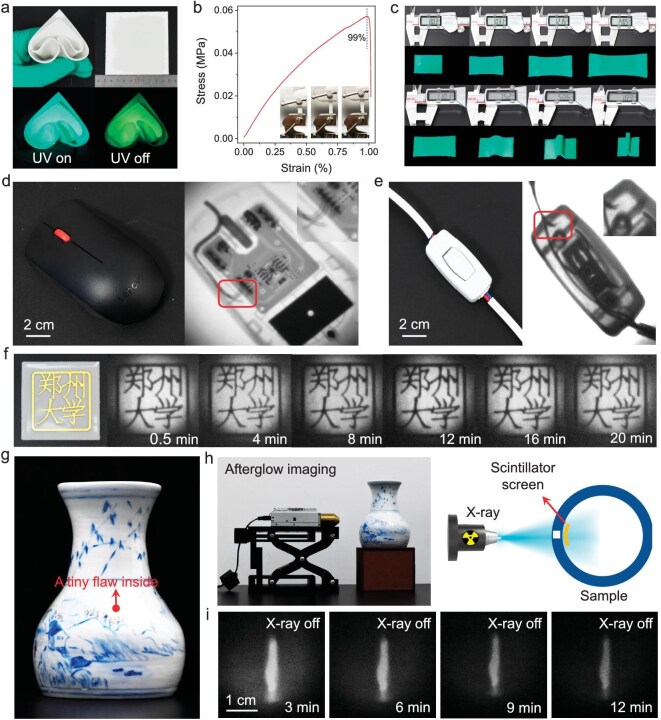
High-resolution X-ray imaging and afterglow imaging using a TPP-3C2B:DMA scintillator screen. (a) Portable large-area flexible scintillator screen. (b) Stress-strain test curve (clamping distance = 15 mm, tensile rate = 2 mm min^−1^, tensile strength = 0.6 MPa). (c) Stretching and bending of the scintillator screen. (d and e) X-ray detection of internal damage for a computer mouse and a circuit switch using the scintillator screen, the flaws are highlighted with red frames. (f) X-ray afterglow imaging of a metal object with a recorded time ranging from 0.5 to 20 min. (g) Photo of a ceramic bottle. (h) Schematic diagram of X-ray afterglow imaging. (i) X-ray afterglow imaging of a tiny flaw inside the bottle.

Portable organic scintillator screens were constructed by combining TPP-3C2B:DMA with polydimethylsiloxane (PDMS) or polyvinylidene fluoride (PVDF), and these screens show excellent scintillation performance (Fig. S34a and Figs S35–S38). The PDMS scintillator screen has excellent foldability, bendability and stretchability, is easy to carry, and can be prepared at different sizes according to the imaging target (Fig. 4a). As shown in Fig. 4b and Fig. S39, stress–strain tests reveal that the maximum tensile deformation of the screen can reach 99% of its own size and that the screen can be cyclically stretched for more than 100 times. In addition, when the scintillator screen stretching/bending cycle is repeated 1000 times, the XEL performance is almost unchanged (Fig. 4c and Fig. S40). The scintillator screen is highly hydrophobic and has a contact angle between the screen surface and water droplets of 117°. Therefore, the XEL intensity of the scintillator screen is unaffected even after it is directly exposed to a large area of water (Fig. S34b and c).

The imaging performance of the scintillator screens was subsequently investigated. As shown in Fig. S34d–i, the resolution of the PDMS scintillator screen can reach 18.3 lp mm^−1^ (27.3 μm), whereas the imaging resolution of the PVDF scintillator screen can reach 24.6 lp mm^−1^ (20.3 μm), which are pretty good values among the reported scintillators (Table S11). Under X-ray irradiation, the internal structure and damage information of a computer mouse and a circuit switch can be recorded on the scintillator screens (Fig. 4d and e).

More importantly, compared with inorganic afterglow scintillator screens, which release stored images at high temperatures [42–44], the organic scintillator screens are the first room-temperature afterglow scintillator screens. As shown in Fig. 4f and Video S3, X-ray can be used to store images on the scintillation screens for up to 20 min. The benefits of the X-ray afterglow imaging enable the detection of flaws inside objects with complex shapes that cannot be detected by direct X-ray imaging technology. For example, a tiny flaw inside a ceramic bottle can be clearly imaged with X-ray afterglow imaging technology. As shown in Fig. 4g and h, a flexible scintillator screen was stuck on the inner surface of the bottle, and X-rays could pass through the bottle wall and reach the scintillator screen. The scintillator screen was then removed from the bottle, and an image of a tiny flaw is clearly shown on the scintillator screen using afterglow (Fig. 4i). In contrast, the flaw is invisible under direct X-ray imaging. A schematic diagram of direct X-ray imaging is shown in Fig. S41. The flexible scintillator screen was attached to the outer surface of the bottle. However, X-rays have difficulty passing through the entire bottle (the intensity of X-rays rapidly decays with increasing travel distance) and reaching the scintillator screen. As a result, regardless of the direction in which the imaging is carried out, the tiny flaw inside the bottle cannot be viewed. This example suggests that X-ray afterglow imaging might be useful in archaeology and identification of cultural relics. Similarly, a scratch on the metal layer inside a metal-plastic composite pipe was successfully detected via X-ray afterglow imaging technology (Fig. S42), indicating the potential of X-ray imaging technology in industrial applications.

## CONCLUSION

In conclusion, a highly stable CS state trap was constructed in a D-A–doped organic scintillator, leading to a unique TADP process under X-ray irradiation. These features allow the organic scintillator to capture and convert high-energy carriers more efficiently, significantly enhancing the efficiency of exciton generation. As a result, an ultrahigh XEL intensity and X-ray afterglow imaging at room temperature are realized. This work demonstrates that improving the capture and conversion of high-energy carriers is a brand-new strategy for the design of high-performance organic scintillators.

## Supplementary Material

nwaf045_Supplemental_Files
