# Supplementary material for: Thermally activated delayed phosphorescence triggered by charge separation state carrier storage in an organic scintillator
Source: Natl Sci Rev. 2025 Feb 12;12(5):nwaf045. doi: 10.1093/nsr/nwaf045 (PMC11970249; doi:10.1093/nsr/nwaf045)
Supplement: nwaf045_Supplemental_Files [file nwaf045_supplemental_files.zip › Supplementary_Materials_for_NSR.pdf]

Charge separation state carrier storage-triggered thermally activated delayed phosphorescence in an organic scintillator

Ruo-Yu Cao[1,#], Yu-Bing Si[1,#], Qi Yang[1,#], Zi-Ying Gao[1,#], Jia-Wang Yuan[1], Yi Zhao[2], Qiu-Chen Peng[1,*], Kai Li[1,*], Shuang-Quan Zang[1,*] and Ben Zhong Tang[3]

[1]Tianjian Laboratory of Advanced Biomedical Sciences, Henan Key Laboratory of Crystalline Molecular Functional Materials, College of Chemistry, Zhengzhou University, Zhengzhou 450001, China

[2]State Key Laboratory of Physical Chemistry of Solid Surfaces, iChEM, Fujian Provincial Key Lab of Theoretical and Computational Chemistry, and College of Chemistry and Chemical Engineering, Xiamen University, Xiamen 361005, China

[3]School of Science and Engineering, Shenzhen Institute of Aggregate Science and Technology, The Chinese University of Hong Kong, Shenzhen (CUHK-Shenzhen), Shenzhen 518172, China

[4] Lead contact

Correspondence: pengqc@zzu.edu.cn

Correspondence: likai@zzu.edu.cn

Correspondence: zangsqzg@zzu.edu.cn

Contents

1. Experimental..........S3

1.1 Reagents..........S3

1.2 Apparatus..........S3

1.3 Synthesis of TPP-3C2B:DMA, TPP-3C2B:DEA and TPP-3C2B:MeDMA.S4

1.4 Synthesis of TPP:CuI, BrTPP:CuI and 3BrTPP:CuI..........S4

1.5 Fabrication of scintillation screens..........S5

1.6 X-ray crystallography..........S6

1.7 Calibration of the radiation dose rate of the X-ray tube..........S6

1.8 XEL measurements..........S8

1.9 Measurement of the X-ray detection limit of the samples..........S8

1.10 Measurement of the LY..........S8

1.11 Optimization and determination of the doping ratio..........S10

1.12 Molecular dynamics (MD) simulations..........S11

1.13 DFTB calculations..........S13

1.14 DFT calculations..........S14

1.15 Hoogenstraaten method for calculating the trap depth..........S14

1.16 T_m-T_{stop} method for calculating the number of traps..........S15

1.17 Photoconductive gain measurements..........S15

1.18 Temperature-dependent radioluminescence measurements..........S15

1.19 X-ray imaging..........S16

1.20 Calculation of the spatial resolution..........S16

1.21 References..........S17

2. Caption of video..........S20

3. Selected spectra and data referred in the paper..........S21

1. Experimental

1.1 Reagents

Unless otherwise stated, all raw materials were commercially available, were of analytical grade, and did not need to be purified. (3-Bromopropyl)triphenylphosphonium bromide and N,N-dimethylaniline were purchased from five companies (Aladdin Reagent Co., Ltd., Shanghai, China; Macklin Biochemical Technology Co., Ltd., Shanghai, China; Sigma-Aldrich Chemical Co., Shanghai, China; TCI Chemical Co., Shanghai, China; and J&K Chemical, Beijing, China). Triphenylphosphine, (4-bromophenyl)diphenylphosphine, tris(4-bromophenyl)phosphane, copper(I) iodide, (S)-(-)-2,2’-bis(diphenylphosphino)-1,1’-binaphthyl N,N-diethylaniline and N,N-dimethyl-p-toluidine were purchased from Alpha Chemical Co., Zhengzhou, China. All other materials, including solvents of analytical grade and chromatographic grade, were purchased from Sinopharm Chemical Reagent Beijing Co., Beijing, China. Commercial scintillators EJ-200, anthracene, 4CzIPN, DMAc-TRz, CaF_2:Eu, $Bi_4Ge_3O_{12}$, and YAP:Ce were purchased from EPIC Crystal Co., Suzhou, China. SYLGARD 184 silicone elastomer (containing a curing agent) was purchased from Alpha Chemical Co., Zhengzhou, China. LiF (diameter, 4.5 mm; thickness, 0.8 mm) and PVDF substrates (diameter 5 mm; aperture, 0.43 μm; thickness, 0.1 mm) were purchased from Guangzhou Rongfan Technology Co., Ltd, Guangzhou, China.

1.2 Apparatus

SCXRD measurements were performed on a Bruker D8 VENTURE diffractometer with Mo-Kα radiation (λ = 0.71073 Å) at 300 K. A Bruker 600 Avance nuclear magnetic resonance (NMR) spectrometer was used to collect NMR data. A Rigaku MiniFlex diffractometer (Cu-Kα; λ = 1.54178 Å; 2θ range of 3-50°) was used to collect PXRD data in air at room temperature. HPLC spectra were collected by an Agilent 1260 Infinite HPLC system with an Agilent ZORBAX Eclipse Plus C18

column. Scanning electron microscopy (SEM) measurements were performed with a Zeiss Sigma 500. A HORIBA FluoroLog-3 fluorescence spectrometer with an integrating sphere ($BaSO_4$) was used to determine the photoluminescence quantum yield (PLQY). XEL and photoluminescence spectra at different temperatures were recorded by an Edinburgh FS5 fibre optic fluorescence spectrometer with a temperature control system (NSTEC HCS621GXY), a xenon lamp source, and a miniature X-ray source (Moxtek Inc.; target material: silver and tungsten). Thermoluminescence spectra were recorded by an LTTL-3DS thermoluminescence spectrometer. The photoconductive gain was measured with a Keysight-B1500A semiconductor parameter analyser. The density of the screens was determined with a G-DenPyc 2900 densitometer. The stress-strain curves and cyclic tensile test data were obtained by a SENS-UTM4103 electronic universal testing machine. The contact angle between a water drop and a screen was measured by a Powereach-JC2000C1 contact angle measuring instrument. Photographs of X-ray or UV-induced luminescence and X-ray imaging were acquired with a digital camera (Nikon D850 coupled with an AF-S Micro-Nikkor 105 mm 2.8G or an AF-S Micro-Nikkor 40 mm 2.8G) in all-manual mode.

1.3 Synthesis of TPP-3C2B:DMA, TPP-3C2B:DEA and TPP-3C2B:MeDMA

The synthesis of TPP-3C2B:DMA was performed according to previous literature[1]. 2 g (4.3 mmol) of TPP-3C2B was dissolved in 50 mL of dichloromethane. Afterwards, 10.75 mL (86 mmol) of DMA was dissolved in 50 mL of ethyl acetate and spread over a dichloromethane solution containing TPP-3C2B. After standing for 24 h, colourless and transparent millimetre-scale crystals of TPP-3C2B:DMA could be obtained with a yield of 90% (based on TPP-3C2B). TPP-3C2B:DEA and TPP-3C2B:MeDMA were synthesized via similar processes, and their yields were 89% and 81% (based on TPP-3C2B), respectively.

1.4 Synthesis of TPP:CuI, BrTPP:CuI and 3BrTPP:CuI

The method for synthesizing TPP:CuI analogues was performed according to the previous literature[2]. BINAP (0.62 g, 1 mol) and CuI (0.19 g, 1 mmol) were mixed in dichloromethane and stirred for 12 h under a nitrogen atmosphere. The resulting mixture, with a large amount of yellow precipitate, was filtered and recrystallized to obtain 0.6 g of BINAP-CuI crystals. Then, TPP:CuI analogues were further prepared through melt-cast methods. TPP derivatives (TPP, BrTPP and 3BrTPP) and 0.1 mol% BINAP-CuI were mixed on quartz sheets and placed on a heating table under nitrogen atmosphere protection. The quartz sheet was then heated to 200 °C until the solid mixture completely melted into an organic liquid. The mixture was held at this temperature for 1 min and then quickly annealed to room temperature. The mixed liquid gradually solidified into a yellow solid at room temperature.

1.5 Fabrication of scintillation screens

2 g of TPP-3C2B was dissolved in 50 mL of dichloromethane, and 10.87 mL of DMA was dissolved in 50 mL of ethyl acetate. The two solutions were then carefully mixed to obtain a stratified solution. The stratified mixture was left to stand for 12 h and then transferred to an ultrasonic cleaner for 20 min. A white powder gradually formed during this process. The white powder deposited at the bottom of the beaker was filtered, and colourless and transparent microcrystals of TPP-3C2B:DMA were obtained with a yield of 80% (based on TPP-3C2B) (Supplementary Fig. 43a). 50 mg of TPP-3C2B:DMA microcrystal was placed in a 7 mL centrifuge tube. 2 mL of n-hexane and 5 small steel balls with a diameter of 3 mm were added. TPP-3C2B:DMA nanocrystals could be obtained by grinding the samples for 3 cycles with a ball mill (Supplementary Fig. 43b). The parameters of each cycle were as follows: ball milling time, 100 s; ball milling frequency, 50 Hz; interval time, 30 s; and number of ball milling times, 60 times. TPP-3C2B:DMA nanocrystals were used to prepare the scintillator screens.

The SYLGARD 184 silicone elastomer and curing agent were premixed at a

mass ratio of 5:1 to form the premixed polymer matrix of PDMS. TPP-3C2B:DMA nanocrystals were then uniformly dispersed in an n-hexane solution, added to the premixed polymer matrix and vigorously stirred to form PDMS micelles. The mass ratio of TPP-3C2B:DMA to the premixed polymer matrix was 1:4. The PDMS micelles were then placed in a Plexiglas mould (5 cm × 5 cm × 0.5 mm or 10 cm × 10 cm × 1 mm) and heated for 3 h at 65 ℃ using a high-precision glue maker for solidification. After the PDMS micelles cooled to room temperature, the sample was peeled off to obtain a smooth and flat PDMS scintillator screen.

100 mg of TPP-3C2B:DMA nanocrystal was uniformly dispersed in 100 mL of ethyl acetate, after which the solution was filtered through a commercial PVDF substrate (diameter, 5 mm; aperture, 0.43 μm; thickness, 0.1 mm) with the aid of a vacuum pump. The PVDF screen was subsequently peeled off to obtain a particle-deposited scintillator screen.

1.6 X-ray crystallography

Data collection and reduction were performed using the APEX3 software package. All the structures were solved via direct methods (SHELXS)[3] and refined via full-matrix least squares on F^2 using OLEX2[4], which uses the SHELXL-2015 module[5]. All the atoms were anisotropically refined. Hydrogen atoms were placed in calculated positions refined using idealized geometries and assigned fixed isotropic displacement parameters. Different strategies were applied for structure refinement according to the electron density distribution. The detailed information of the crystal data and refinement results are summarized in Supplementary Tables 1-4.

1.7 Calibration of the radiation dose rate of the X-ray tube

First, 50 mg **1** was dissolved in 10 mL DCM, and then 10 g silica gel was added. After mixing well, the DCM was removed under reduced pressure.

To confirm the reliability of the experiment, the radiation dose rates generated by the X-ray tube under different currents, voltages and distances were calibrated

considering the distance between the sample and the X-ray tube during the test. In this calibration method, commercial LiF dosimeters, which were consistently selected, were fixed at a distance *d* (mm) directly in front of the exit of the X-ray tube, and the LiF dosimeters were irradiated using the X-ray tube after different tube voltages, *U* (kV), tube currents, *I* (μA), and irradiation times, *t* (min), were set. After the irradiation was complete, the LiF dosimeters were placed in a thermoluminescence spectrometer to test the dose received by the LiF dosimeters. The measurement was repeated 5 times for each set of parameters, and the average value was taken to obtain the dose. Finally, the X-ray dose rate at the current voltage, current and detection distance was obtained by dividing the dose by the irradiation time. The specific calibration method is as follows:

1. Determine the dose rate of the X-ray tube at different voltages: Fix the detection position (10 mm) and the current of the X-ray tube (80 μA), and set 5 different voltage values (15 kV, 20 kV, 30 kV, 40 kV, and 50 kV). After the voltage of the X-ray tube is changed, the corresponding dose rate is recorded and analysed to determine the relationship between the voltage and the dose rate (Supplementary Fig.6a).

2. Determine the dose rate of the X-ray tube at different currents: Fix the detection position (10 mm) and the voltage of the X-ray tube (50 kV), and set 5 different current values (10 μA, 20 μA, 40 μA, 60 μA, and 80 μA). After the current of the X-ray tube is changed, the corresponding dose rate is recorded and analysed to determine the relationship between the current and the dose rate (Supplementary Fig. 6b).

3. Determine the dose rate of the X-ray tube at different distances: Fix the current (80 μA) and voltage (50 kV) of the X-ray tube, and set 5 different distance values (10 mm, 20 mm, 30 mm, 40 mm, and 50 mm). After the distance is changed, the corresponding dose rate is recorded and analysed to determine the relationship

between the distance and the dose rate (Supplementary Fig. 6c).

4. Based on the experimental data, a fitting formula describing the change in the dose rate ($\dot{D}$) with the current (I), voltage (U) and distance (d) is established:

$$\dot{D}(U,I,d)=\frac{6.62\times(-5.23+0.638I)\times(62.34-62.45\times\exp(\frac{15-U}{25.84}))}{(d+7.47)^2} \qquad (2)$$

As shown in Supplementary Fig. 6d, the X-ray tube and the sample are fixed in a lead box, and the distance between the X-ray tube and the sample is 10 mm. Thus, according to the equation, the corresponding $\dot{D}$ for different combinations of I, U at a distance of 10 mm can be calculated (Supplementary Fig. 6e).

1.8 XEL measurements

The sample was pressed into identical sheets by an infrared mould, which resulted in the same surface area and thickness. All the measurements were carried out under the same X-ray source, and all the settings, such as the slit and temperature, were the same. Based on this method, the XEL spectra of the samples were compared.

1.9 Measurement of the X-ray detection limit of the samples

XEL spectra of the scintillator were obtained with various X-ray doses from 0.27 to 45.79 mGy min^{-1}. The X-ray radiation dose was controlled by changing the current and voltage of the miniature X-ray source. The relationships of the current and voltage of the miniature X-ray source with the corresponding radiation dose are shown in Supplementary Fig. 6e. The LOD was calculated as LOD = $3\sigma/k$, where σ is the standard deviation calculated based on repeated tests of background signals ten times and k is the slope of the linear fitting curve.

1.10 Measurement of the LY

Commercial scintillator materials with known LY were chosen as the standard samples. Both the standard samples and the samples to be measured were prepared into wafers with the same specific surface area and thickness using a tablet press.

The X-ray attenuation coefficients for different thicknesses of the samples were

calculated based on the X-ray absorption curves fitted to the photon cross-section database as well as the density information obtained from crystallographic cell calculations (the attenuation of X-ray by air was considered to be zero). The radioluminescence spectra of different samples with the same thickness under the same X-ray irradiation were obtained by using a fluorescence spectrometer equipped with an X-ray tube. Then, the XEL intensity of the standard sample and the sample to be measured could be obtained by integrating the spectra.

To compare the XEL intensities of two samples after the same X-ray energy is absorbed, the XEL intensity should be normalized to the same X-ray attenuation. The relative LY of the sample to be measured can be calculated by taking the ratio of the normalized XEL intensity and the LY of the standard sample. The specific calculation method is as follows:

The X-ray attenuation coefficient (α) should first be calculated via equation (3):

$$\alpha = c(\varepsilon)\times\rho \qquad (3)$$

where $c(\varepsilon)$ is the photon cross-section function obtained from the XCOM web database (https://www.nist.gov/pml/xcom-photon-cross-sections-database), ε is the corresponding X-ray photon energy (keV), and ρ is the density of the scintillator.

The X-ray attenuation efficiency (AE) is calculated via equation (4):

$$AE(\varepsilon,d) = (1-\mathrm{e}^{-c(\varepsilon)\rho d})\times 100\% \qquad (4)$$

where d is the thickness of the scintillator.

The X-ray attenuation efficiency versus the scintillator thickness ($AE(d)$) for the entire X-ray photon energy range (from 0 to 50 keV) is obtained via equation (5):

$$AE(d) = \int_0^{50} \frac{AE(\varepsilon,d)\times R(\varepsilon)d\varepsilon}{\left(\int_0^{50} R(\varepsilon)d\varepsilon\right)} \qquad (5)$$

where $R(\varepsilon)$ is the X-ray output spectrum of our tube at 50 kV, and the unit of ε is keV.

The XEL spectra were tested under the same conditions, and the corresponding

photon counts (XEL_{measured}) were obtained by integrating the XEL spectra. The normalized emissive photon count ($XEL_{\text{normalized}}$) was calculated via equation (6):

$$XEL_{\text{normalized}} = \frac{XEL_{\text{measured}}}{AE(d)} \qquad (6)$$

The LY of the TPP-3C2B:DMA crystal can be calculated via equation (7):

$$LY_{\text{sample}} = LY_{\text{standard}} \times \frac{XEL_{\text{normalized}}(\text{sample})}{XEL_{\text{normalized}}(\text{standard})} \qquad (7)$$

$XEL_{\text{normalized}}$ (standard) represents the emissive photon count of the standard scintillator normalized to the respective X-ray attenuation efficiency at a thickness of 0.4 mm. which can be found in the literature[6].

1.11 Optimization and determination of the doping ratio

The influences of the doping ratio of DMA in TPP-3C2B:DMA on the luminescence efficiency and afterglow time were carefully investigated (Supplementary Fig. 44). First, the DMA feeding ratio (molar ratio of DMA and TPP-3C2B) used in the synthesis of TPP-3C2B:DMA was adjusted to obtain different TPP-3C2B:DMA samples. The DMA feeding ratio was chosen to range from 2.5 to 25. If the DMA feeding ratio is too low (< 2.5), then the luminescence of the resulting crystals is very weak; if the DMA feeding ratio is too high (> 25), then high-quality single crystals cannot form, which will affect the luminescence intensity of the doped system. Moreover, to ensure the reliability of the experimental data, powder crystals with different feeding ratios were prepared as wafers with the same thickness and specific surface area (Supplementary Fig. 44a). The XEL spectra of the samples were measured (Supplementary Fig. 44b). As shown in Supplementary Fig. 44c, the XEL intensity of the samples gradually increases with increasing DMA feeding ratio. When the DMA feeding ratio reaches 20, the sample has the highest XEL intensity. When the DMA feeding ratio is greater than 25, the quality of the grown crystals deteriorates, and the XEL intensity significantly decreases. In addition, as shown in Supplementary Fig. 44d, the DMA feeding ratio has almost no effect on the afterglow time of TPP-3C2B:DMA. Based on above results, a DMA feeding ratio of 20 was

used for the synthesis of TPP-3C2B:DMA. As shown in Supplementary Fig. 44e, the X-ray excited afterglow spectra of TPP-3C2B:DMA with a DMA feeding ratio of 20 can be clearly recorded even after 7 h.

Moreover, to determine the doping ratio of DMA in TPP-3C2B:DMA (DMA feeding ratio of 20), an HPLC test was carried out. The HPLC data are shown in Supplementary Fig. 11, and the concentration of DMA in the TPP-3C2B:DMA solution (c_{DMA}) was calculated as 0.01053 g L^{-1} according to the following equation (8):

$$c_{\mathrm{x}} = c_{\mathrm{R}} \times \frac{A_{\mathrm{x}}/V_{\mathrm{x}}}{A_{\mathrm{R}}/V_{\mathrm{R}}} \qquad (8)$$

where c_{x} is the concentration of the doped substance in the sample solution (i.e., the concentration of DMA in the TPP-3C2B:DMA solution), c_{R} is the concentration of the reference solution (i.e., the concentration of DMA in the reference solution), A_{x} is the area of the peak corresponding to the doped substance in the sample solution, A_{R} is the peak area for the reference solution, V_{x} is the injection volume of the sample solution, and V_{R} is the injection volume of the reference solution.

By combining $c_{\mathrm{DMA}}$with the concentration of the TPP-3C2B:DMA solution ($c_{\text{TPP-3C2B:DMA}}$), the molar doping ratio of DMA in TPP-3C2B:DMA (x_{DMA}) can be calculated as 0.4974% according to equation (9):

$$\begin{aligned} x_{\mathrm{DMA}} &= \frac{c_{\mathrm{DMA}}/M_{\mathrm{DMA}}}{c_{\text{TPP-3C2B:DMA}}/M_{\text{TPP-3C2B:DMA}}} \times 100\% \\ &\approx \frac{c_{\mathrm{DMA}}/M_{\mathrm{DMA}}}{c_{\text{TPP-3C2B:DMA}}/M_{\text{TPP-3C2B}}} \times 100\% \qquad (9) \end{aligned}$$

In addition, to further rule out the effect of impurities on the optical performance of TPP-3C2B:DMA, chemical reagents purchased from different commercial sources were used to prepare TPP-3C2B:DMA (Supplementary Fig. 4). As shown in Supplementary Fig. 4a, TPP-3C2B and DMA purchased from five different commercial sources were cross-combined to produce 25 sets of samples. The experimental results show that the XEL and afterglow behaviours are identical for all

samples (Supplementary Fig. 4b-c), suggesting that potential impurities have little effect on the luminescence properties of TPP-3C2B:DMA.

1.12 Molecular dynamics (MD) simulations

This study aimed to improve our comprehension of the photophysical characteristics of the organic scintillator. To achieve this goal, a series of multiscale theoretical simulations were conducted, including MD simulations, density functional theory-based tight-binding (DFTB) calculations, and density functional theory (DFT) calculations.

Given the lack of a crystal structure for the TPP-3C2B complex, the initial task was to confirm whether DMA adsorbs onto or inserts into the TPP-3C2B crystal. To explore this, a TPP-3C2B (001) surface was constructed to study the adsorption behaviour of DMA. In the insertion model, a TPP-3C2B molecule on the TPP-3C2B (001) surface was replaced by DMA (Supplementary Fig. 45). The TPP-3C2B and DMA monomers were optimized using the Gaussian 16 package at the B3LYP/def2-SVP leve[7]. The single-point energy was subsequently calculated at the B3LYP/def2-TZVP level. The GAFF force field topology files for TPP-3C2B and DMA were generated using the acpepy script[8]. MD simulations were performed using the GROMACS software package (version 2019.6)[9]. The total potential energy was calculated as the sum of the valence terms, including bond stretching, angle bending, torsion, and nonbonded interactions. Nonbonded interactions were described by the Lennard-Jones potential, and van der Waals interactions between different atom species followed standard geometric mean combination rules.

The simulation process began with energy minimization of the initial configurations via the steepest descent method. This was followed by a 200 ps MD simulation under the NVT ensemble to pre-equilibrate the system, with a time step of 2 fs. The temperature was linearly increased from 0 to 298 K within 200 ps and then maintained at 298 K throughout the rest of the simulation. A 50 ns production

simulation was then carried out. In all the simulations, the temperature was held constant at 298 K via the V-rescale thermostat algorithm[10]. The bond lengths were constrained by the LINCS algorithm, and periodic boundary conditions were applied in all directions[11]. Short-range nonbonded interactions were cut off at 1.0 nm, with long-range electrostatics calculated via the particle mesh Ewald method[12]. The trajectories were recorded every 2 ps and visualized using VMD 1.9.3 and VideoMach 5.15.1[13].

Additionally, to compare the results, two DMA derivatives, DEA and MeDMA, were simulated using the insertion model in place of DMA (Supplementary Fig. 46).

1.13 DFTB calculations

To evaluate the cytotoxicity of **2**, CCK-8 assays were used. A549 cells were seeded in a 96-well plate at a density of 6000-8000 cells per well. After cell growth for 12 h, the medium in each well was replaced with 100 mL fresh medium containing different concentrations of **2**. Then, some of the samples were treated with white light irradiation (5 mW/cm^2, 10 min) and further incubated for 24 h. After that, 10 μL CCK-8 solution was added to each well. After incubation for 1 h, the absorption of each well (450 nm) was recorded *via* a Perkin-Elmer Victor3™ plate reader.

To confirm the alterations in the unit cell parameters after doping, DFTB calculations were conducted using the PWmat package and a modified version of the DFTB+ software[14-15]. These calculations utilized cuSOLVER routines instead of the MAGMA library[16] to enhance the computational performance. All the DFTB3 calculations included Lennard-Jones dispersion correction[17] and employed the 3ob-3-1 Slater-Koster set of parameters[18]. The self-consistent charge convergence criterion was set to 10^{-6} elementary charges, and geometry optimization was performed using a rational function optimizer with a force threshold criterion of 2×10^{-4} Hartree/Bohr.

According to the DFTB-based calculations, for the undoped crystal of

TPP-3C2B, the total volume is 93419.324552 Å^3, whereas for the doped crystal, in which one TPP-3C2B is replaced by a DMA molecule, the optimized total volume is 93381.064941 Å^3. The lattice parameters obtained via theoretical calculations and experiments are shown in Supplementary Table 12 and are almost the same. Owing to the negligible change in the lattice parameters, as shown in Supplementary Fig. 47, observing changes in the PXRD signals after doping is experimentally difficult. Moreover, SCXRD data of TPP-3C2B:DMA with different DMA feeding ratios were also collected, and these data barely showed any differences (Supplementary Tables 1-4).

1.14 DFT calculations

The inherent flexibility of the scintillator monomer was addressed by employing a two-layer ONIOM approach to accurately evaluate the electronic structures within the crystal matrix. In this framework, a specific cluster at the core of the crystal was treated as the "QM layer" using the PBE0/def2-SVP level of theory. Simultaneously, the surrounding molecules were designated as the "MM layer" and were analysed using the universal force field (UFF). This approach was augmented by an electronic embedding technique that directly incorporated the partial charges from the MM region into the QM Hamiltonian[19].

Based on the MD simulations, DMA clearly has weak interactions with two adjacent TPP-3C2B molecules (Supplementary Fig. 48). Therefore, when examining the doped system of TPP-3C2B, the ONIOM model was utilized considering two TPP-3C2B molecules and one DMA molecule within the QM region, along with an additional 20 TPP-3C2B molecules in the MM region. To construct the diabatic state of triplet states localized on the donor or acceptor, constrained density functional theory (CDFT) was employed[20], utilizing the same theoretical level of PBE0/def2-SVP in Q-Chem software[21]. Additionally, the spin-orbit coupling matrix elements were determined via ORCA 5.0.3 software, which relies on the PBE0

functional and the DKH-def2-TZVP basis set[22]. Complementary to these calculations, Mulliken charge and spin population analyses were conducted, with the results visualized via the Multiwfn program[23-24].

1.15 Hoogenstraaten method for calculating the trap depth

TL bands spanning the temperature range from 275 to 425 K with different heating rates ranging from 0.5 to 10 K s^{-1} were recorded. When the heating rate (β_h) decreases, the peak temperature (T_m) shifts to the low-temperature side. The trap depth (ε) of TPP-3C2B:DMA can be calculated via equation (10):

$$\frac{\beta_h \times \varepsilon}{k_B \times T_m^2} = s \times \exp\left(\frac{-\varepsilon}{k_B \times T_m}\right) \quad (10)$$

where k_B is the Boltzmann constant and s is the frequency factor. Equation (10) can be written as equation (11):

$$\ln\frac{T_m^2}{\beta_h} = \varepsilon \times \frac{1}{k_B \times T_m} \times \ln\frac{\varepsilon}{s \times k_B} \quad (11)$$

Thus, the trap depth (i.e., the slope of the fitting line) can be determined by plotting $\ln\frac{T_m^2}{\beta_h}$ against $\frac{1}{k_B \times T_m}$, which is 0.557 eV (Fig. 3g).

1.16 T_m-T_{stop} method for calculating the number of traps

The T_m-T_{stop} curves were measured according to a previously reported method[25-27]. The sample was irradiated by X-ray for 60 s at 273 K, then heated to a certain preannealing temperature (T_{stop}) and maintained for 5 s. Afterwards, the sample was rapidly quenched to 273 K, and a TL curve was recorded at a heating rate of 2 K s^{-1} in the temperature range from 273 to 425 K. Then, the maximum temperature (T_m) of the peak on the TL curve was recorded. The above measurements were repeated with different preannealing temperatures, and a T_m-T_{stop} curve was obtained.

1.17 Photoconductive gain measurements

The photoconductive gain was measured by using a semiconductor parameter analyser and a miniature X-ray tube. Current-voltage curves were measured in the dark, under UV light and under X-ray irradiation (tube voltage: 50 kV, tube current

dose rate: 100 μA). To fix the crystal and connect the probes well for the measurements, colloidal silver paste drops were placed on opposite sides of the crystal as electrodes. The relevant experimental equipment is shown in Supplementary Fig. 49.

1.18 Temperature-dependent radioluminescence measurements

Temperature-dependent radioluminescence measurements were carried out by connecting an INSTEC temperature control system, X-ray tubes and fluorescence spectrometers with a special liquid light guide. The relevant experimental equipment is shown in Supplementary Fig. 50.

1.19 X-ray imaging

The X-ray imaging system consisted of a miniature X-ray source, a scintillator screen, and a digital camera. During the imaging process, the sample was placed between the X-ray source and scintillator screen, and a digital camera was used to collect pictures from the other side of the scintillator screen. In X-ray afterglow imaging, the X-ray source can be removed after the X-ray has irradiated the object. A photo of the device is shown in Supplementary Fig. 51. During imaging, the environment temperature was 25 ℃, and the distance between the X-ray phototube and the sample was 10 cm. In addition, in the normal X-ray imaging process, the voltage of the X-ray phototube was 50 kV, the current was 100 μA, the sensitivity of the camera was 5000, the aperture size was 4, and the exposure time was 1 s. In the afterglow imaging process, the voltage of the X-ray phototube was 60 kV, the current was 150 μA, the sensitivity of the camera was 12500, the aperture size was 4, and the exposure time was 20 s.

1.20 Calculation of the spatial resolution

According to the literature, the spatial resolution can be determined by using the modulation transfer function (MTF), which represents the transfer ability of the input signal modulation of a spatial frequency (in line pair per millimetre; lp mm-1) relative to its output6. A spatial frequency of MTF = 0.2 is considered to be the spatial resolution limit, as this is the spatial frequency at which an observer cannot visually differentiate between features (although this is subject to observer bias). In this work, the slanted-edge method was used to calculate the MTF curve. First, a standard line-pair mask with a sharp edge was placed on the scintillator, and an image of its edge profile was obtained via an X-ray imaging system. Based on the slanted-edge profile from X-ray imaging, the edge spread function (ESF(x)) was derived. Then, the ESF(x) was differentiated to obtain the line spread function (LSF(x)). After Fourier transformation, the MTF can be calculated according to the following equation (12):

$$\mathrm{MTF}(\nu) = \mathcal{F}(\mathrm{LSF}(x)) = \mathcal{F}\left(\frac{\mathrm{dESF}(x)}{\mathrm{d}x}\right) \qquad (12)$$

where ν is the spatial frequency and x is the position of the pixels. The calculation can be performed via ImageJ software.

1.21 References

1 Alam P, Leung N L C and Liu J et al. Two are better than one: a design principle for ultralong-persistent luminescence of pure organics. *Adv Mater* 2020; **32**: 2001026.

2 Liang X, Luo X-F and Yan Z-P *et al.* Organic long persistent luminescence through in situ generation of cuprous(I) ion pairs in ionic solids. *Angew Chem Int Ed* 2021; **60**: 24437-24442.

3 Sheldrick G M. A short history of SHELX. *Acta Cryst A* 2007; **64**: 112-122.

4 Dolomanov O V, Bourhis L J and Gildea R J *et al.* OLEX2: a complete structure solution, refinement and analysis program. *J Appl Cryst* 2009; **42**: 339-341.

5 Sheldrick G M. Crystal structure refinement with SHELXL. *Acta Cryst C* 2015; **71**: 3-8.

6 Ma W, Su Y and Zhang Q *et al.* Thermally activated delayed fluorescence (TADF) organic molecules for efficient X-ray scintillation and imaging. *Nat Mater* 2022; **21**: 210-216.

7 Frisch M, Trucks G and Schlegel H *et al.* Gaussian 16 revision A. 03.

8 Wang J, Wolf R M and Caldwell J W *et al.* Development and testing of a general amber force field. *J Comput Chem* 2004; **25**: 1157-1174.

9 Abraham M J, Murtola T and Schulz R *et al.* GROMACS: High performance molecular simulations through multi-level parallelism from laptops to supercomputers. *SoftwareX* 2015; **1-2**: 19-25.

10 Bussi G, Donadio D and Parrinello M. Canonical sampling through velocity rescaling. *J Chem Phys* 2007; **126**: 014101.

11 Hess B, Bekker H and Berendsen H J C *et al.* LINCS: a linear constraint solver for molecular simulations. *J Comput Chem* 1997; **18**: 1463-1472.

12 Darden T, York D and Pedersen L. Particle mesh Ewald: An N·log(N) method for Ewald sums in large systems. *J Chem Phys* 1993; **98**: 10089-10092.

13 Humphrey W, Dalke A and Schulten K. VMD: Visual molecular dynamics. *J Mol Graphics* 1996; **14**: 33-38.

14 Hourahine B, Aradi B and Blum V *et al.* DFTB+, a software package for efficient approximate density functional theory based atomistic simulations. *J Chem Phys* 2020; **152**: 124101.

15 Jia W, Fu J and Cao Z *et al.* Fast plane wave density functional theory molecular dynamics calculations on multi-GPU machines. *J Comput Phys* 2013; **251**: 102-115.

16 Tomov S, Dongarra J and Baboulin M. Towards dense linear algebra for hybrid GPU accelerated manycore systems. *Parallel Comput* 2010; **36**: 232-240.

17 Zhechkov L, Heine T and Patchkovskii S *et al.* An efficient a posteriori treatment for dispersion interaction in density-functional-based tight binding. *J Chem Theory Comput* 2005; **1**: 841-847.

18 Lu X, Gaus M and Elstner M *et al.* Parametrization of DFTB3/3OB for magnesium and zinc for chemical and biological applications. *J Phys Chem B* 2015; **119**: 1062-1082.

19 Dapprich S, Komáromi I and Byun K S *et al.* A new ONIOM implementation in Gaussian98. Part I. The calculation of energies, gradients, vibrational frequencies and electric field derivatives1Dedicated to Professor Keiji Morokuma in celebration of his 65th birthday.1. *J Mol Struct Theochem* 1999; **462**: 1-21.

20 Wu Q and Van Voorhis T. Direct calculation of electron transfer parameters through constrained density functional theory. *J Phys Chem A* 2006; **110**: 9212-9218.

21 Shao Y, Gan Z and Epifanovsky E *et al.* Advances in molecular quantum chemistry contained in the Q-Chem 4 program package. *Mol Phys* 2014; **113**: 184-215.

22 Neese F. Software update: The ORCA program system—Version 5.0. *WIREs Comput Mol Sci* 2022; **12**: e1606.

23 Lu T. A comprehensive electron wavefunction analysis toolbox for chemists, Multiwfn. *J Chem Phys* 2024; **161**: 082503.

24 Lu T and Chen F. Multiwfn: A multifunctional wavefunction analyzer. *J Comput Chem* 2012; **33**: 580-592.

25 Barad A, Topaksu M and Hakami J *et al.* Thermoluminescence kinetics in beta-irradiated novel $ZnGa_2O_4:Eu^{3+}$ phosphor produced via gel combustion synthesis. *Ceram Int* 2024; **50**: 11458-11468.

26 Altowyan A S, Sonsuz M and Kaynar Ü H *et al.* Thermoluminescence kinetic parameters of beta irradiated the zinc gallate phosphor using different methods. *Ceram Int* 2023; **49**: 23732-23742.

27 McKeever S W S. On the analysis of complex thermoluminescence. glow-curves: resolution into individual peaks. *Phys Stat Sol (A)* 1980; **62**: 331-340.

2. Caption of video

Video 1. Afterglow of TPP-3C2B:DMA under X-ray excitation.

Video 2. Afterglow of TPP-3C2B:DMA under UV light excitation.

Video 3. X-ray afterglow imaging of a metal object.

3. Selected spectra and data referred in the paper

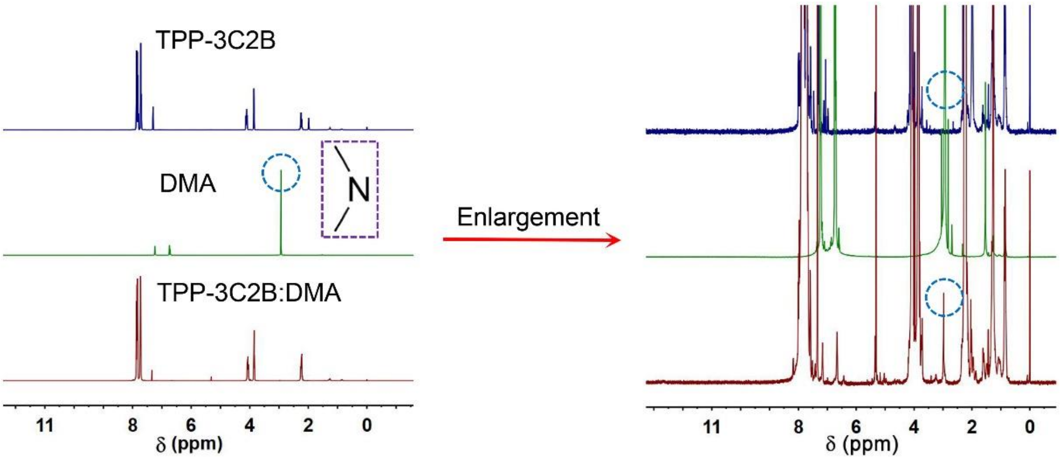

Supplementary Fig. 1 ^{1}H-NMR spectra of TPP-3C2B, DMA and TPP-3C2B:DMA.

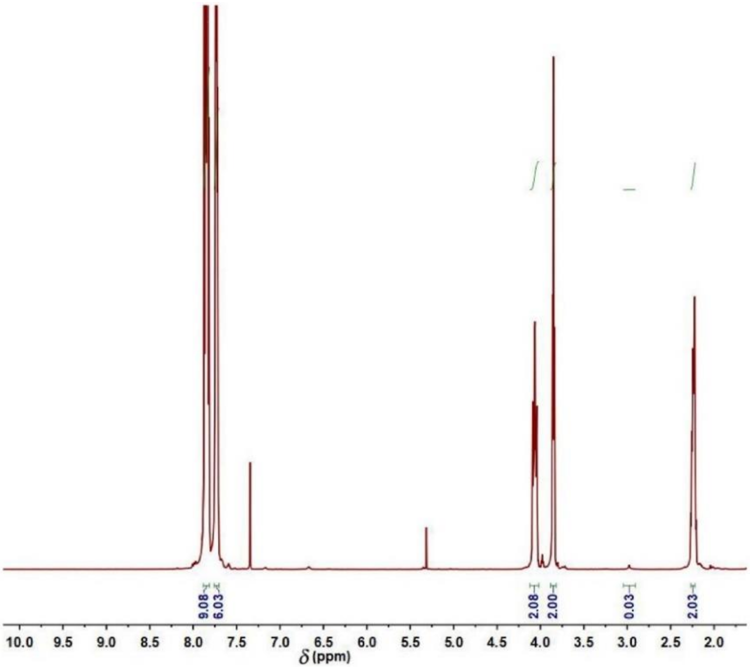

Supplementary Fig. 2 ^{1}H-NMR spectra of TPP-3C2B:DMA.

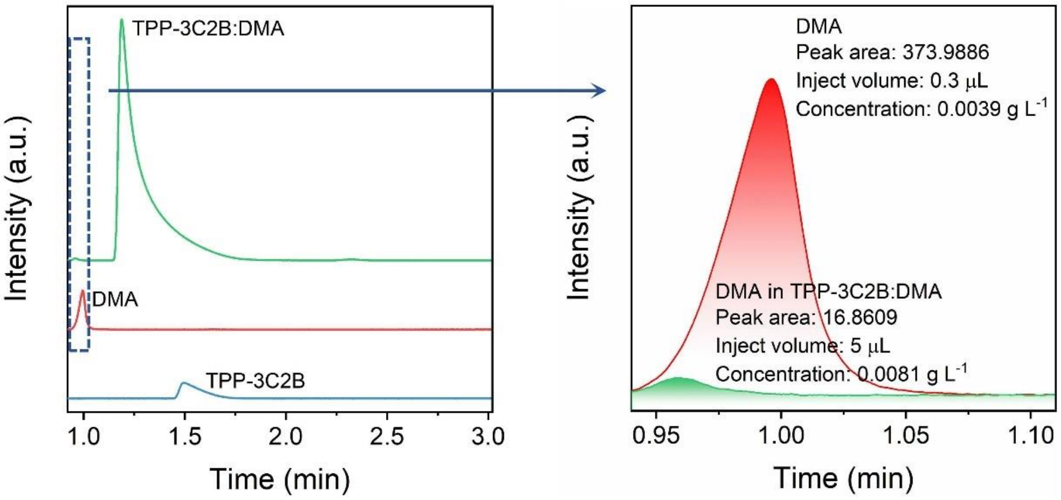

Supplementary Fig. 3 Left: HPLC curves of TPP-3C2B:DMA and the reference solutions. Right: Fractionated gain of the HPLC curves.

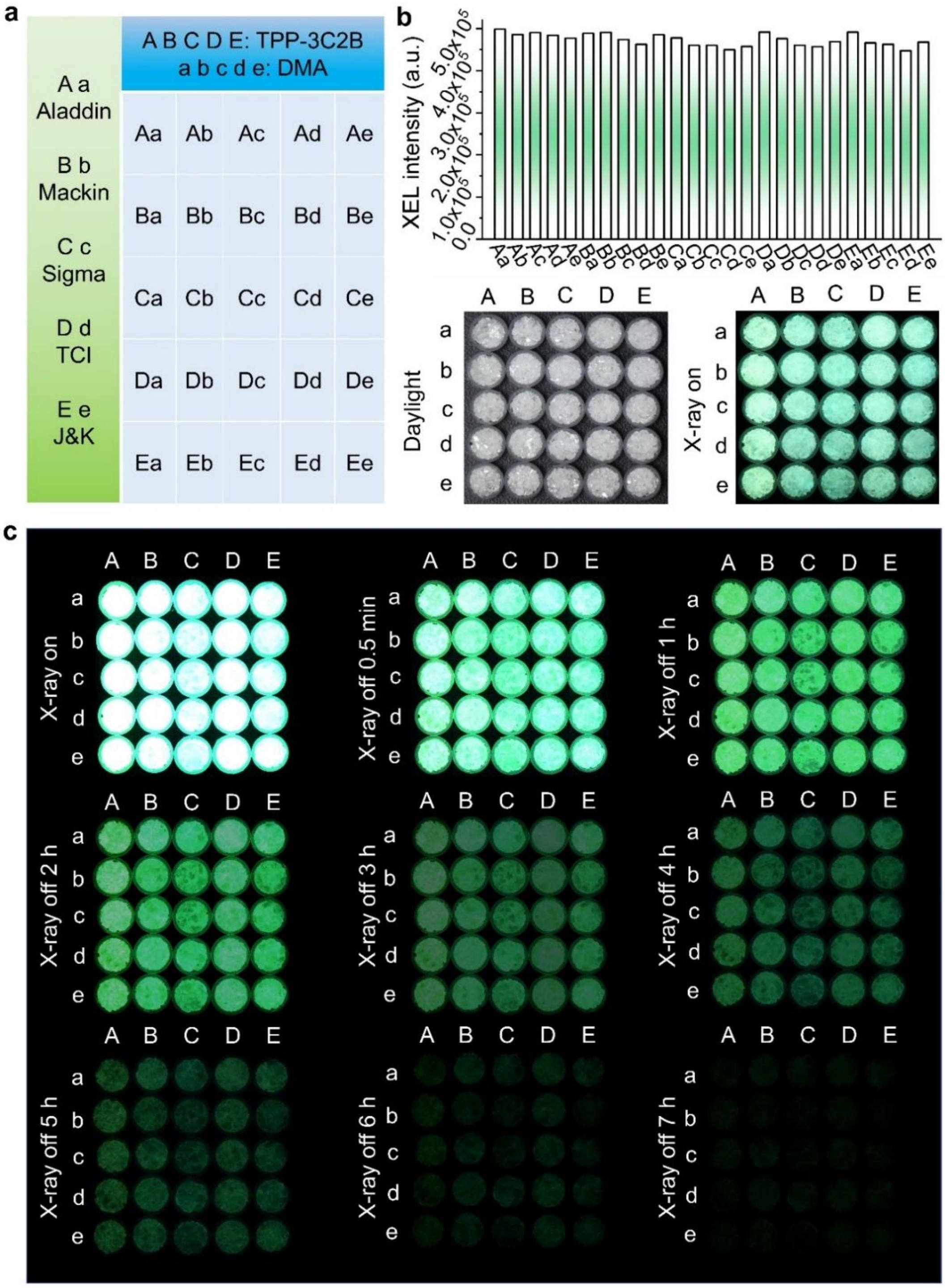

Supplementary Fig. 4 a, Combinations for the 25 sets of samples. **b,** XEL intensities of the samples and corresponding photos under daylight and X-ray conditions. **c,** Photos of X-ray excited afterglow of the samples.

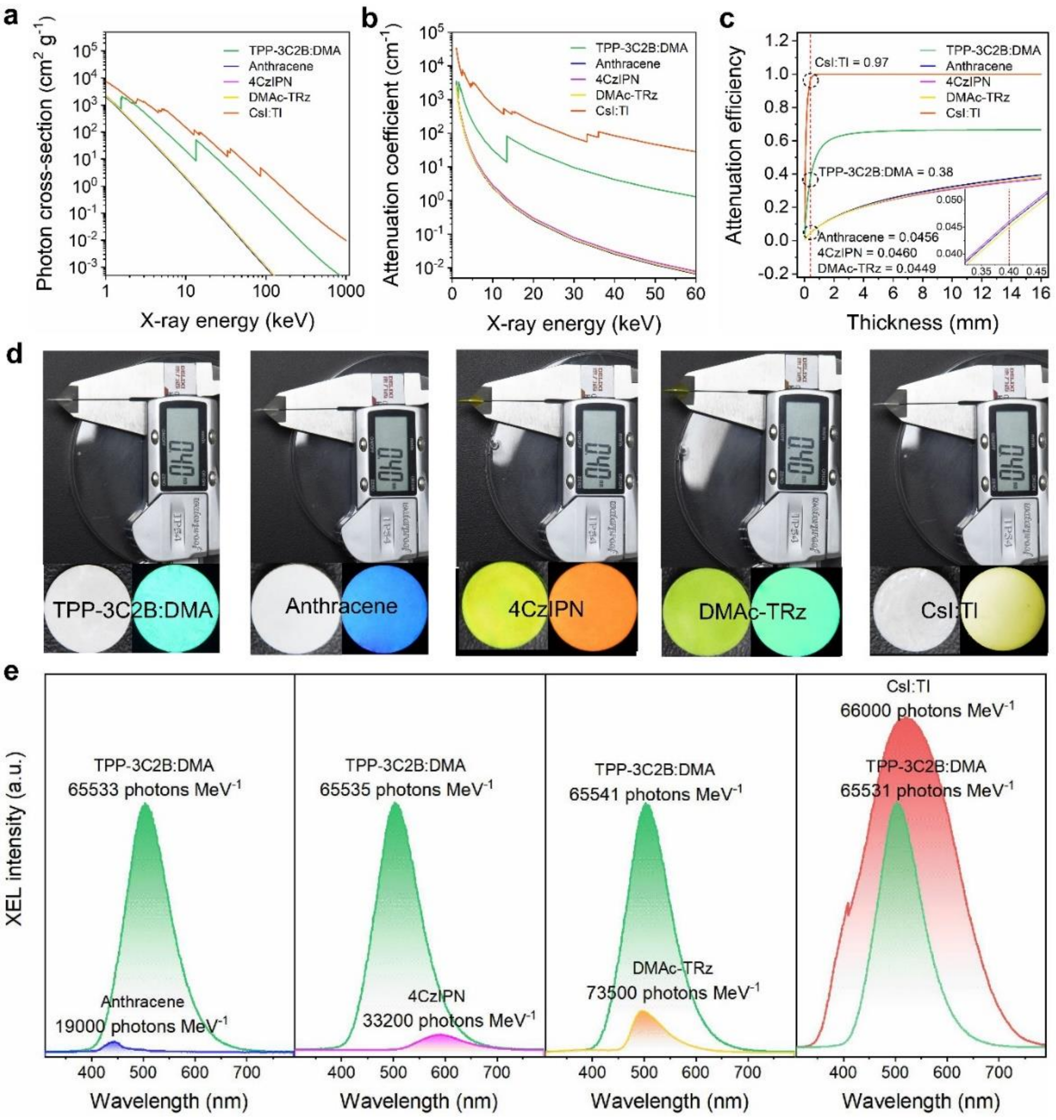

Supplementary Fig. 5 a, Variation in the photon cross-section with the X-ray energy. **b,** Variation in the X-ray attenuation coefficient with the X-ray energy. **c,** Variation in the X-ray attenuation efficiency with the thickness. **d,** Photos of sample thickness measurements and samples (left: under daylight; right: under X-ray). **e,** XEL spectra of TPP-3C2B:DMA and different standard scintillators with the same thickness and cross-sectional area.

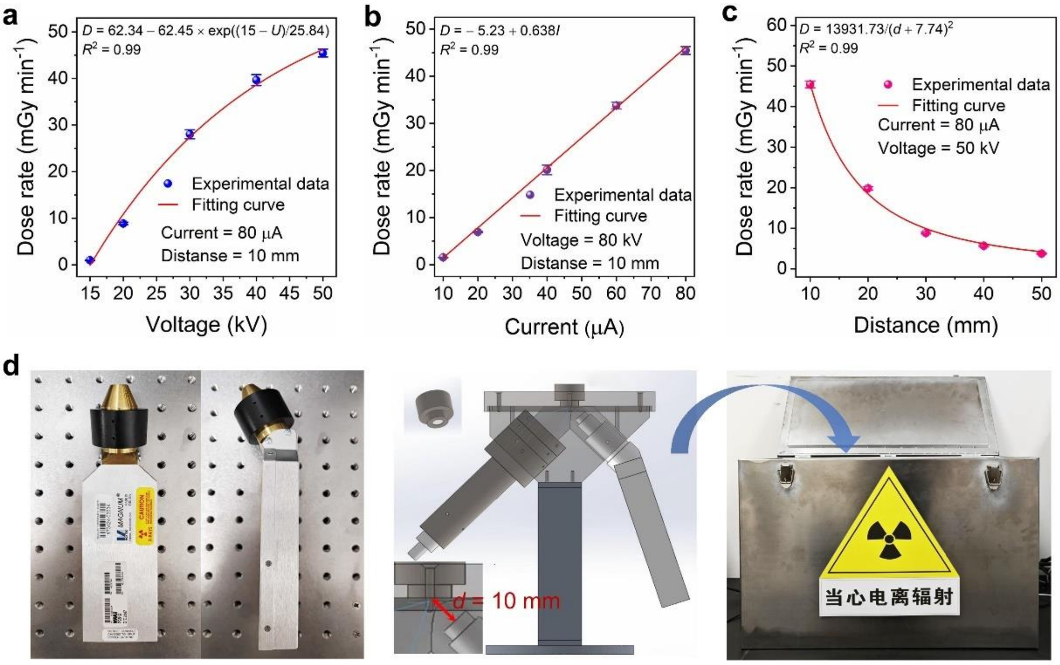

e

Voltage (kV)	20	30	40	50	50	50	50	50	50	50	50	50
Current (μA)	10	10	10	10	15	20	30	40	50	60	70	80
Distance (mm)	10	10	10	10	10	10	10	10	10	10	10	10
Dose rate (mGy min^{-1})	0.27	0.68	0.96	1.15	4.34	7.52	13.90	20.28	26.66	33.04	39.41	45.79
Fitted equation	$\dot{D}(U, I, d) = \frac{6.62 \times (-5.23 + 0.638I) \times (62.34 - 62.45 \times \exp\left(\frac{15 - U}{25.84}\right))}{(d + 7.74)^2}$											

Supplementary Fig. 6 a-c, Dose rate of an X-ray tube at different (a) voltages, (b) currents, and (c) distances. **d,** XEL measurement system. **e,** Corresponding $\dot{D}$ values for different combinations of I and U at a distance of 10 mm.

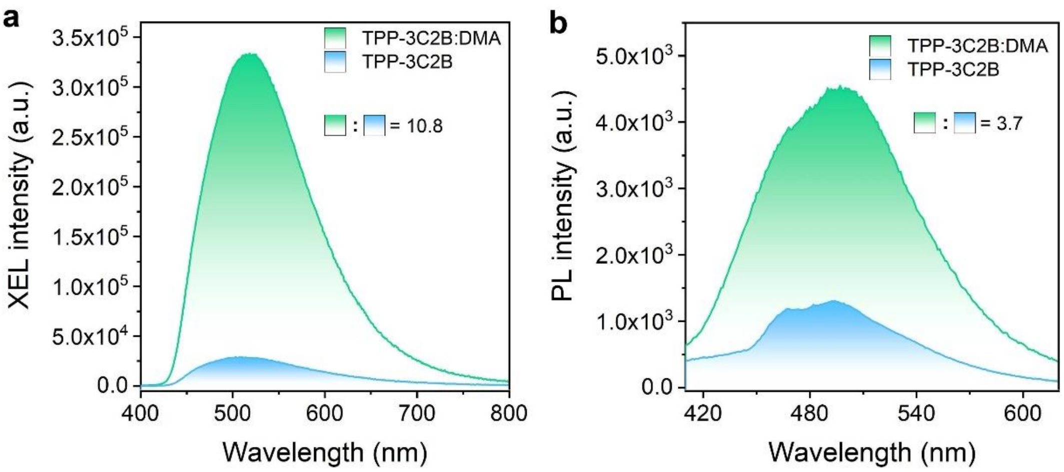

Supplementary Fig. 7 a-b, (a) XEL spectra and **(b)** photoluminescence spectra of TPP-3C2B:DMA and TPP-3C2B with the same thickness and cross-sectional area (thickness = 0.4 mm). The test temperature was 298 K. The integration area ratios were shown inside.

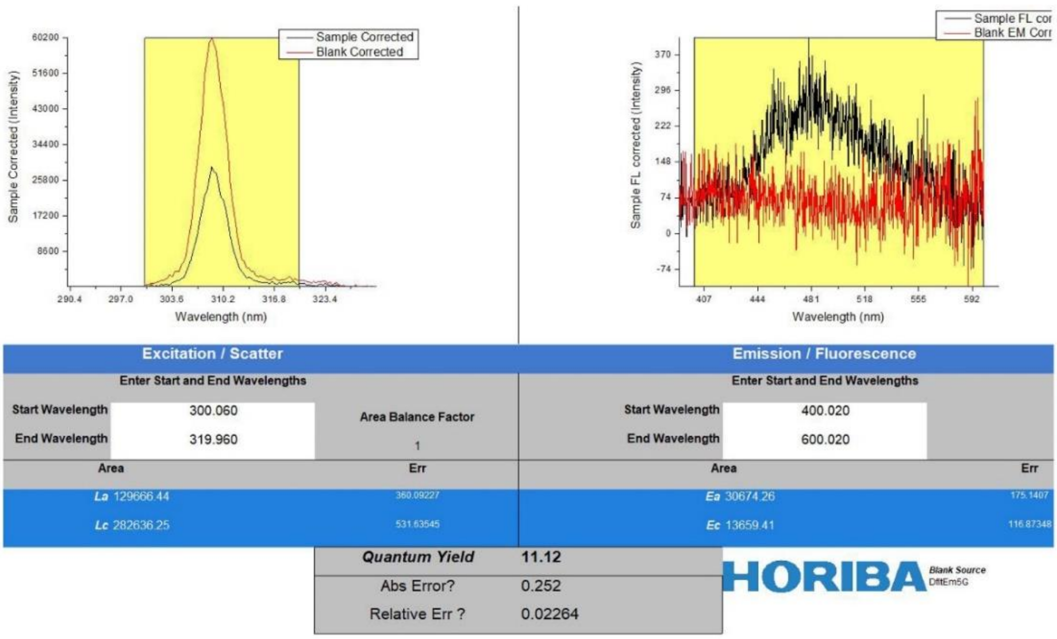

Supplementary Fig. 8 Photoluminescence quantum yield (PLQY) of TPP-3C2B.

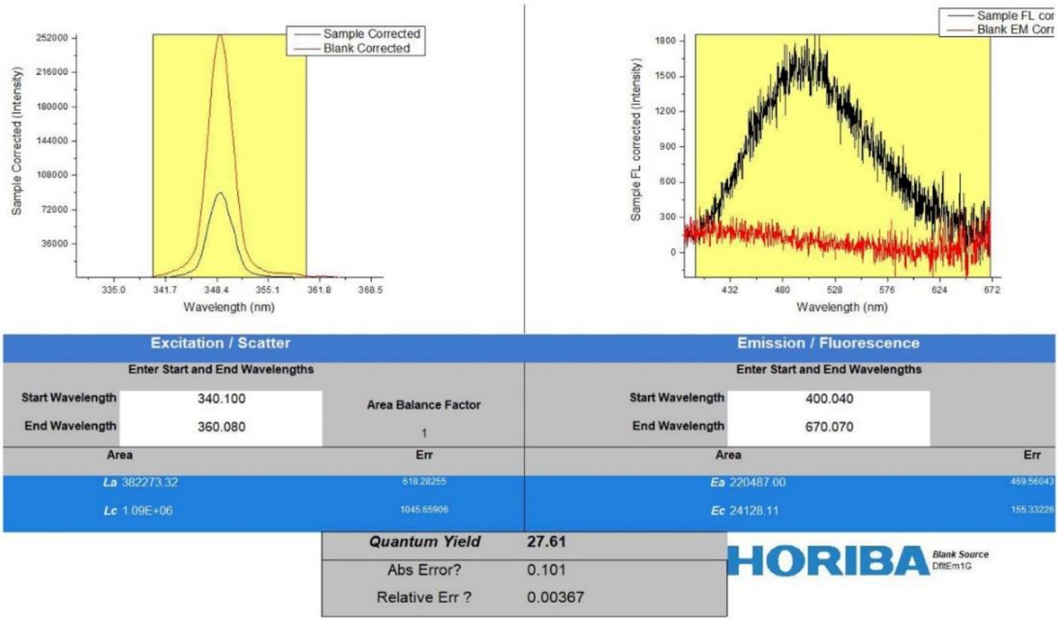

Supplementary Fig. 9 PLQY of TPP-3C2B:DMA.

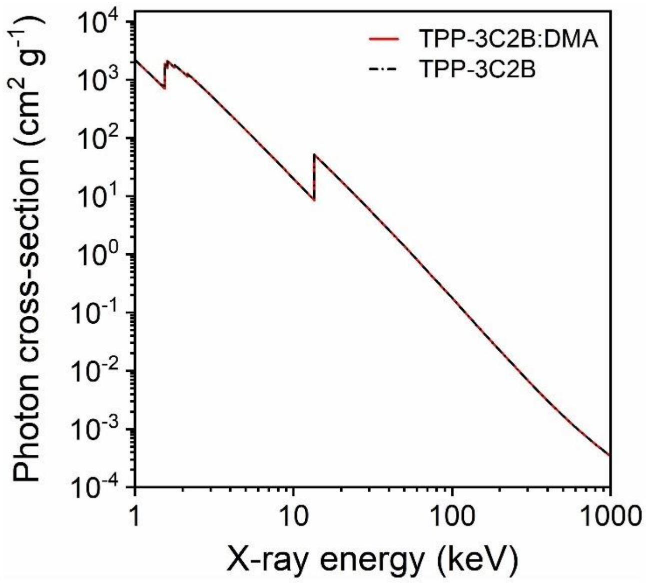

Supplementary Fig. 10 Variation in the photon cross-section with the X-ray energy of TPP-3C2B:DMA and TPP-3C2B.

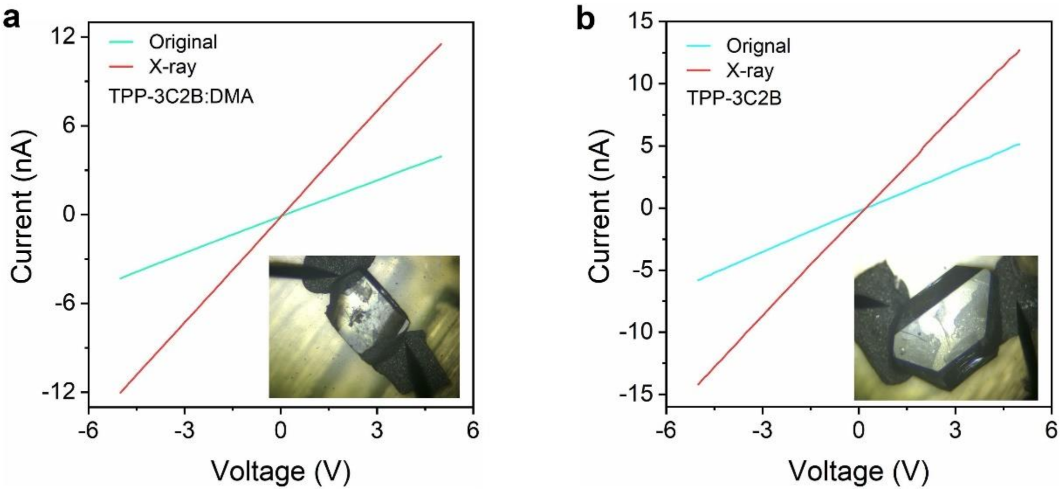

Supplementary Fig. 11 a-b, Current-voltage curves of **(a)** TPP-3C2B:DMA and **(b)** TPP-3C2B in the dark and under X-ray irradiation.

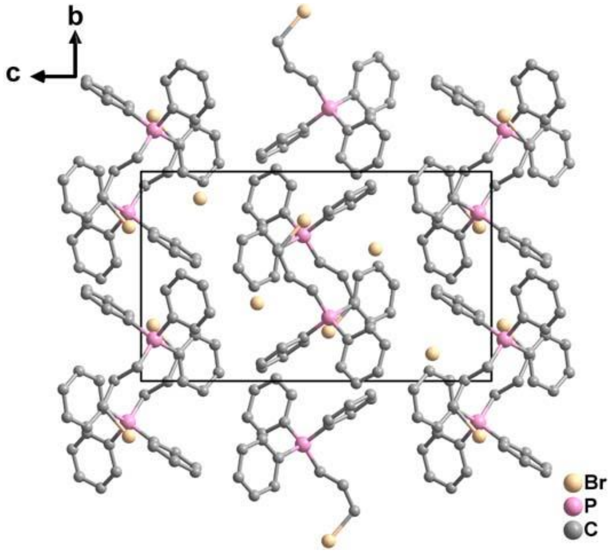

Supplementary Fig. 12 Single-crystal structure of TPP-3C2B.

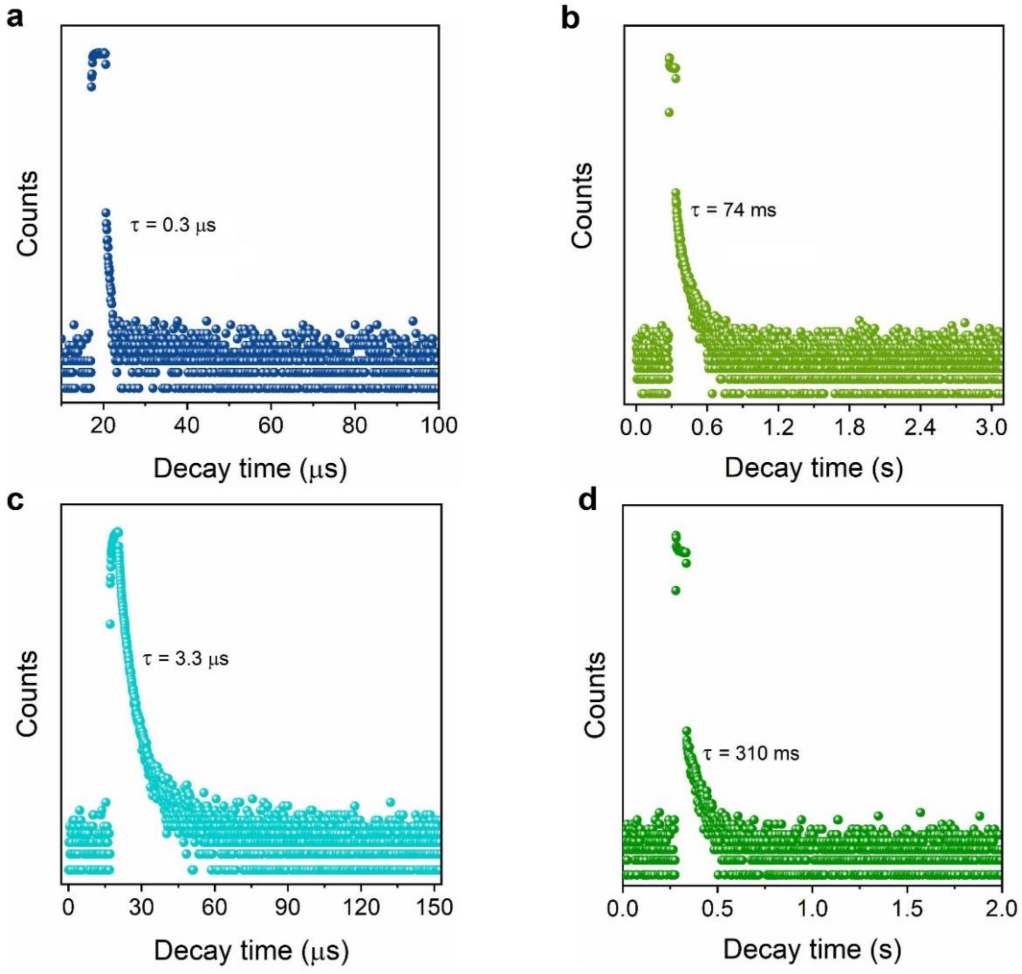

Supplementary Fig. 13 a-d, Luminescence lifetime decay curves of **(a-b)** TPP-3C2B and **(c-d)** TPP-3C2B:DMA.

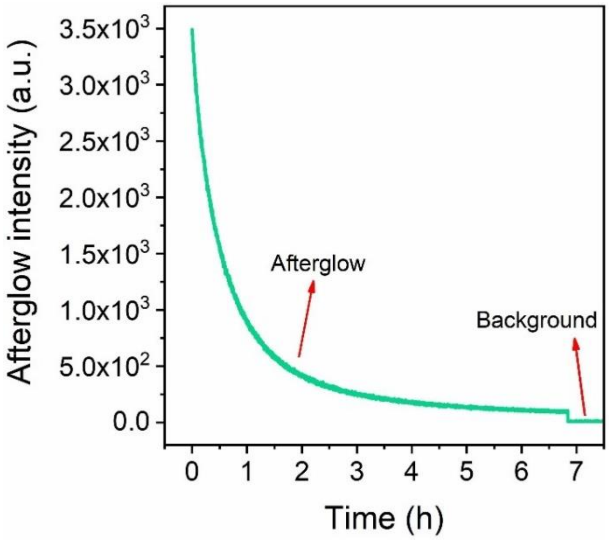

Supplementary Fig. 14 Afterglow profile of TPP-3C2B:DMA after UV light excitation.

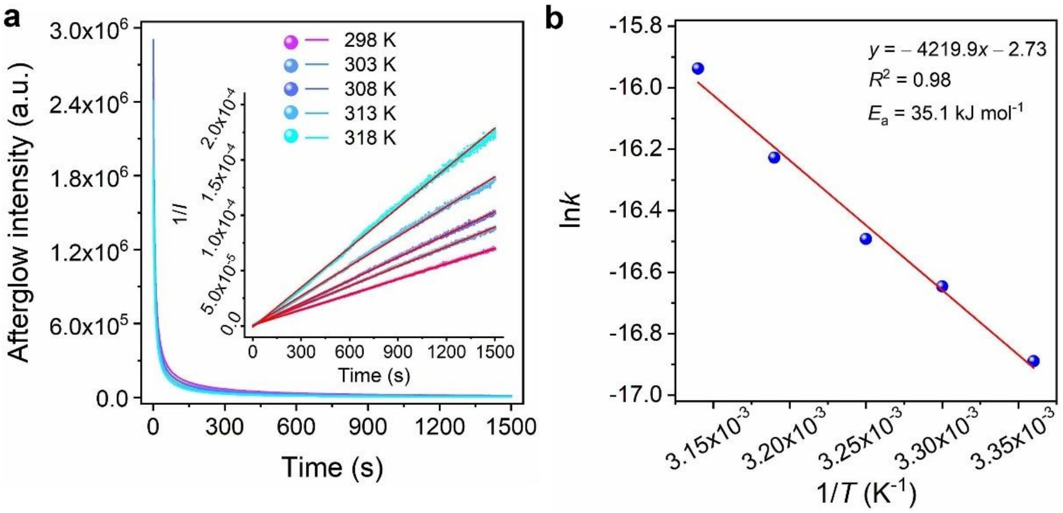

Supplementary Fig. 15 a, Time-dependent emission decay curves of TPP-3C2B:DMA at different temperatures after UV light excitation was stopped. Inset: The curves were fitted with second-order reaction dynamics to calculate the rate constant. **b,** Rate constants of TPP-3C2B:DMA fitted with Arrhenius expressions.

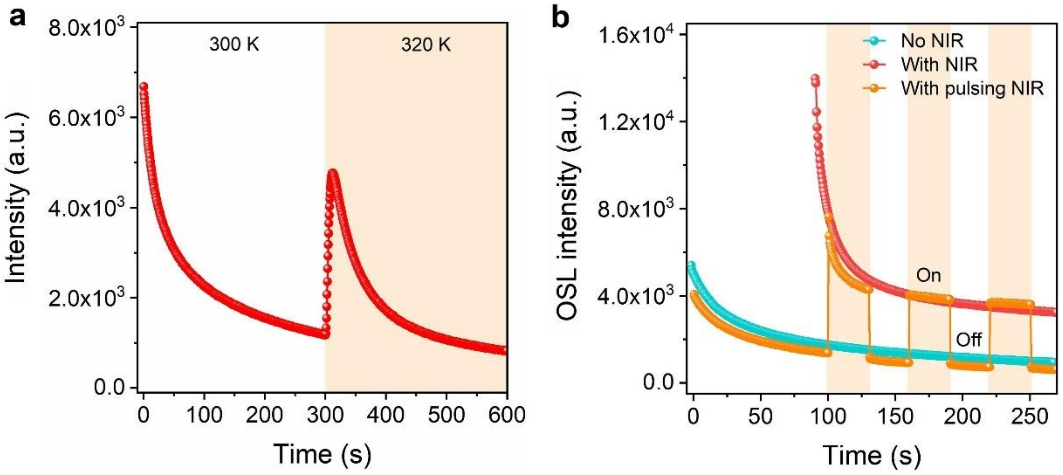

Supplementary Fig. 16 a, Afterglow of TPP-3C2B:DMA under different thermal stimulation conditions. Conditions: X-ray excited TPP-3C2B:DMA was maintained at 300 K for 5 min and then heated to 320 K at a heating rate of 20 K s^{-1}. **b,** Decay curves of TPP-3C2B:DMA with 980 nm NIR photostimulation in continuous output mode (red line), pulsed mode (repeating on and off every 30 s, orange line), and without NIR photostimulation (cyan line).

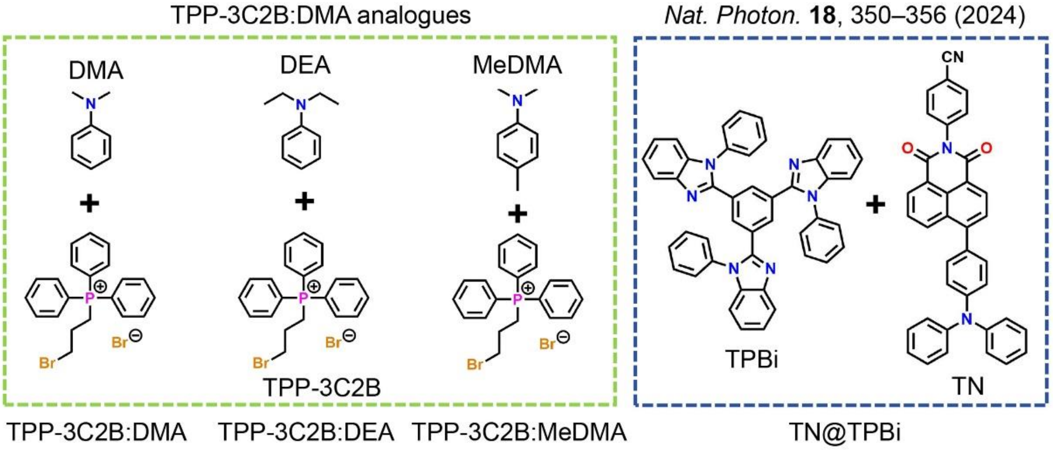

Supplementary Fig. 17 Compositions of TPP-3C2B:DMA and the control compounds TPP-3C2B:DEA, TPP-3C2B:MeDMA and TN@TPBi.

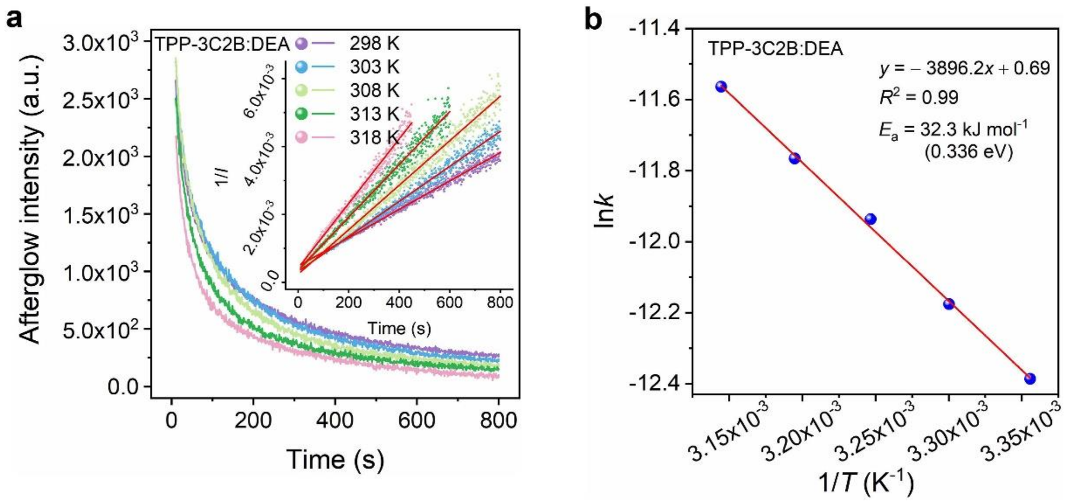

Supplementary Fig. 18 a, Time-dependent emission decay curves of TPP-3C2B:DEA at different temperatures after X-ray excitation was stopped. Inset: The curves were fitted with second-order reaction dynamics to calculate the rate constant. **b,** Rate constants of TPP-3C2B:DEA fitted with Arrhenius expressions.

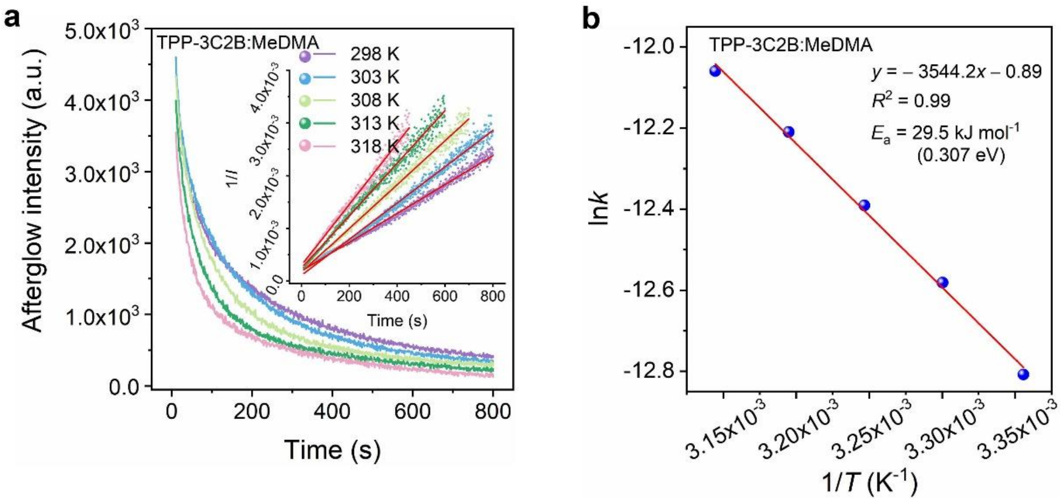

Supplementary Fig. 19 a, Time-dependent emission decay curves of TPP-3C2B:MeDMA at different temperatures after X-ray excitation was stopped. Inset: The curves were fitted with second-order reaction dynamics to calculate the rate constant. **b,** Rate constants of TPP-3C2B: MeDMA fitted with Arrhenius expressions.

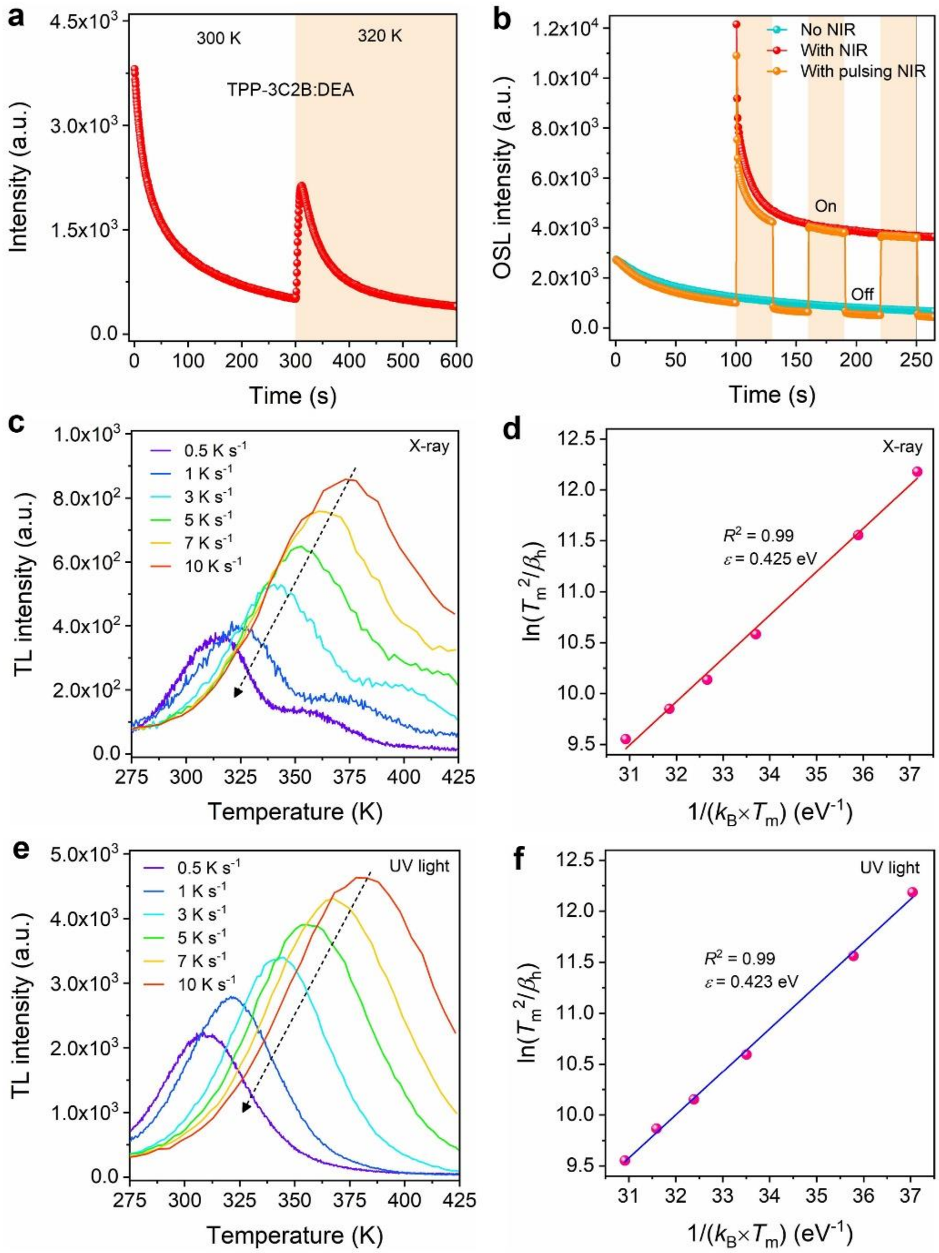

Supplementary Fig. 20 a, Afterglow of TPP-3C2B:DEA under different thermal stimulation conditions. Conditions: X-ray excited TPP-3C2B:DEA was maintained at 300 K for 5 min and then heated to 320 K at a heating rate of 20 K s^{-1}. **b,** Decay curves of TPP-3C2B:DEA with 980 nm NIR photostimulation in continuous output mode (red line) and in pulsed mode (repeating on and off every 30 s, orange line) and without NIR photostimulation (cyan line). **c,** TL curves of TPP-3C2B:DEA after X-ray excitation under different heating rates. **d,** Estimation of the trap depth via the Hoogenstraaten method based on the X-ray excited TL data. **e,** TL curves of TPP-3C2B:DEA after UV light excitation under different heating rates. **f,** Estimation of the trap depth via the Hoogenstraaten method based on the UV light excited TL

data.

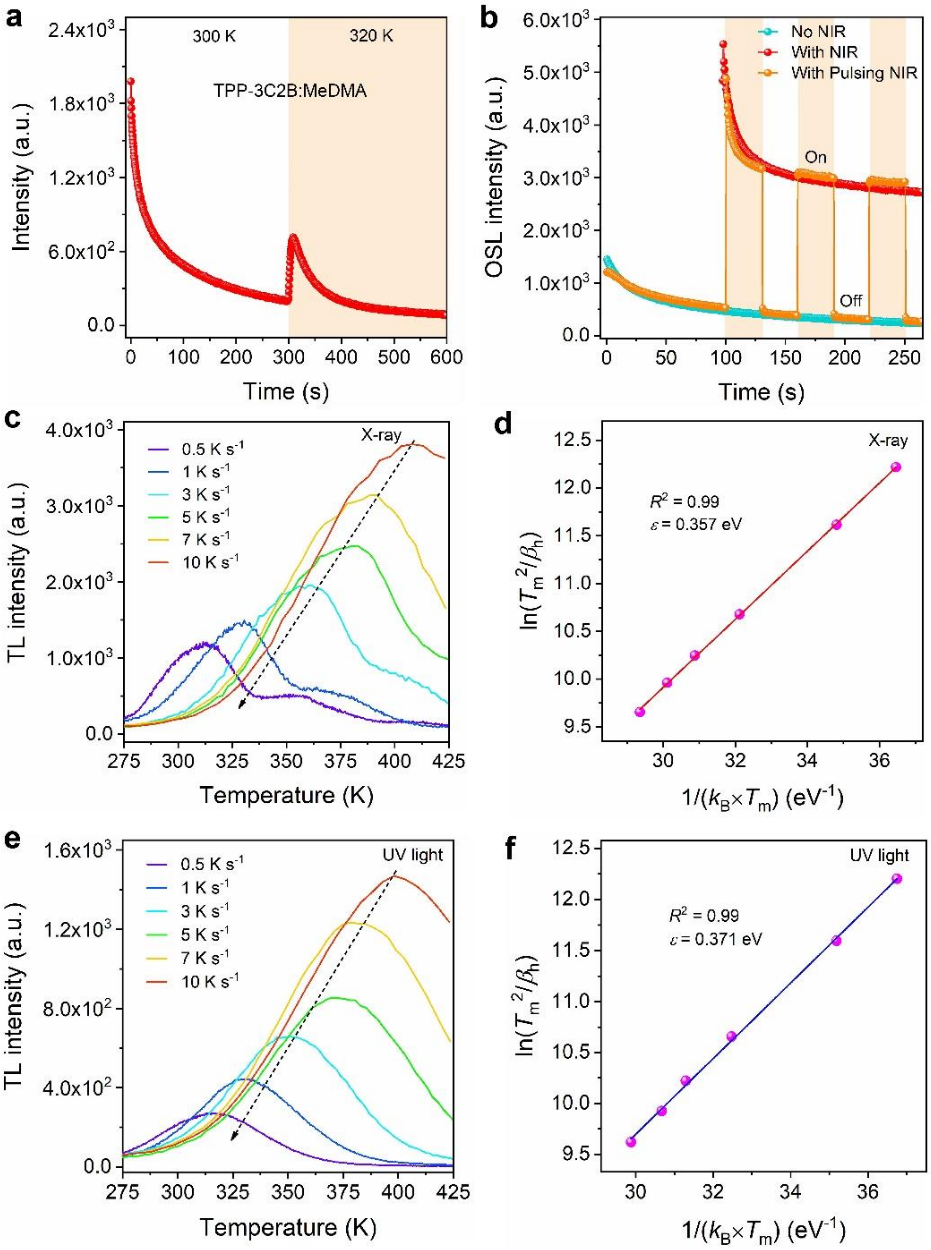

Supplementary Fig. 21 a, Afterglow of TPP-3C2B:MeDMA under different thermal stimulation conditions. Conditions: X-ray excited TPP-3C2B: MeDMA was maintained at 300 K for 5 min and then heated to 320 K at a heating rate of 20 K s^{-1}. **b,** Decay curves of TPP-3C2B:MeDMA with 980 nm NIR photostimulation in continuous output mode (red line) and in pulsed mode (repeating on and off every 30 s, orange line) and without NIR photostimulation (cyan line). **c,** TL curves of TPP-3C2B:MeDMA after X-ray excitation under different heating rates. **d,** Estimation of the trap depth via the Hoogenstraaten method based on the X-ray excited TL data. **e,** TL curves of TPP-3C2B:MeDMA after UV light excitation under

different heating rates. **f,** Estimation of the trap depth via the Hoogenstraaten method based on the UV light excited TL data.

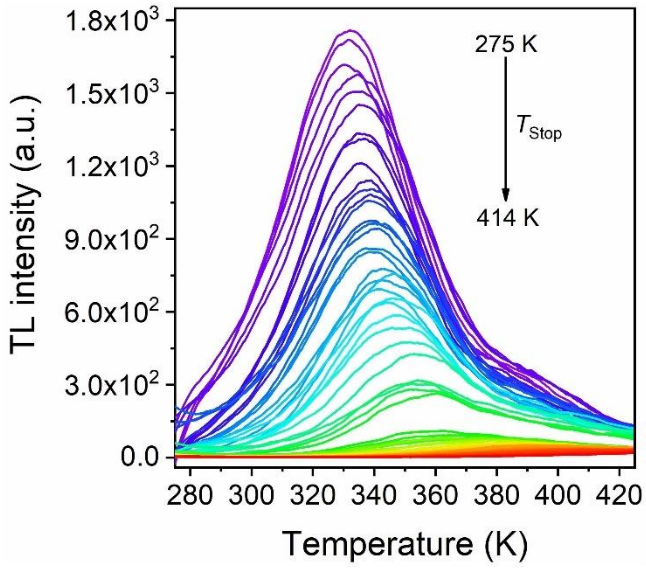

Supplementary Fig. 22 TL curves of TPP-3C2B:DMA for different T_{stop} values. T_{stop} is the preannealing temperature.

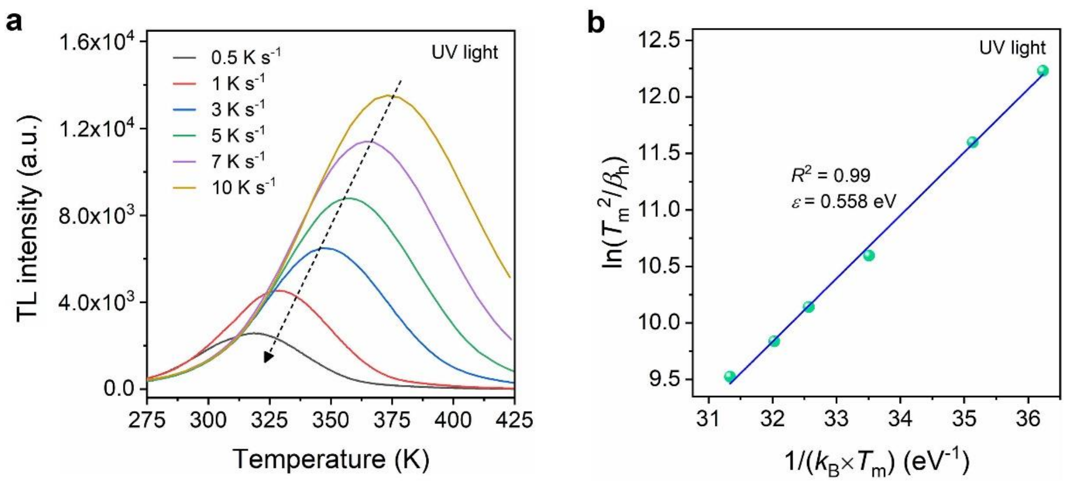

Supplementary Fig. 23 a, TL curves of TPP-3C2B:DMA after UV light excitation under different heating rates. **b,** Estimation of the trap depth via the Hoogenstraaten method based on the UV light excited TL data.

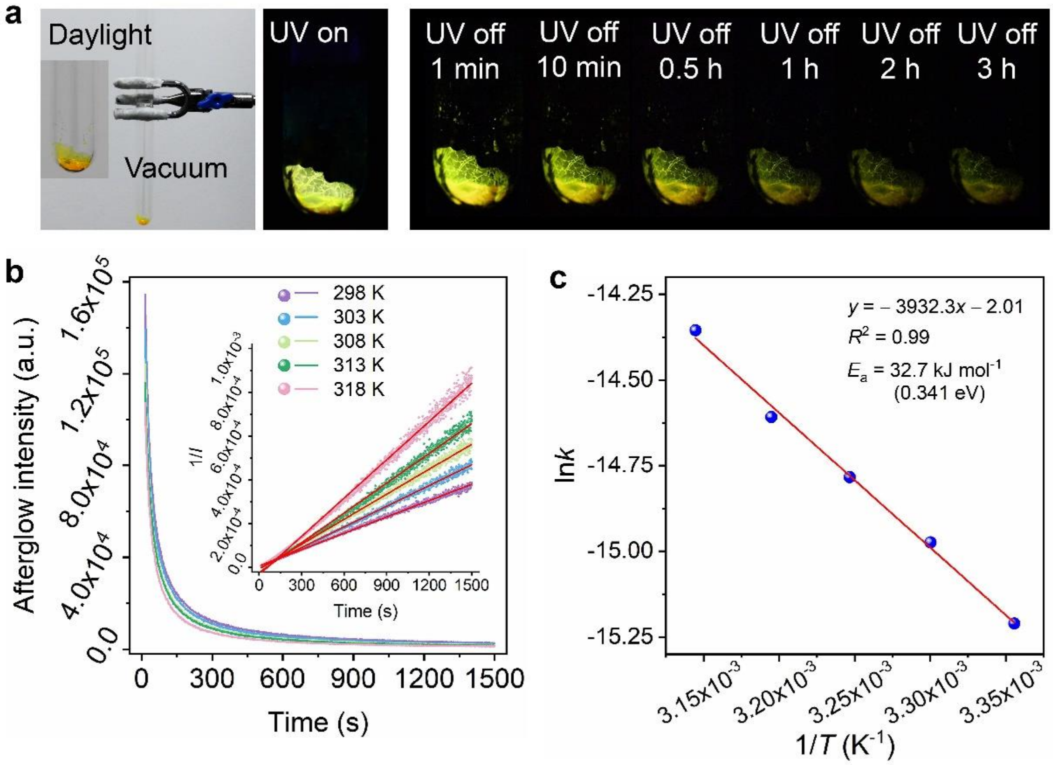

Supplementary Fig. 24 a, Photos of TN@TPBi under daylight and UV light and its afterglow. **b,** Time-dependent emission decay curves of TN@TPBi at different temperatures after UV light excitation was stopped. Inset: The curves were fitted with second-order reaction dynamics to calculate the rate constant. **c,** Rate constants of TN@TPBi at different temperatures fitted with the Arrhenius expression.

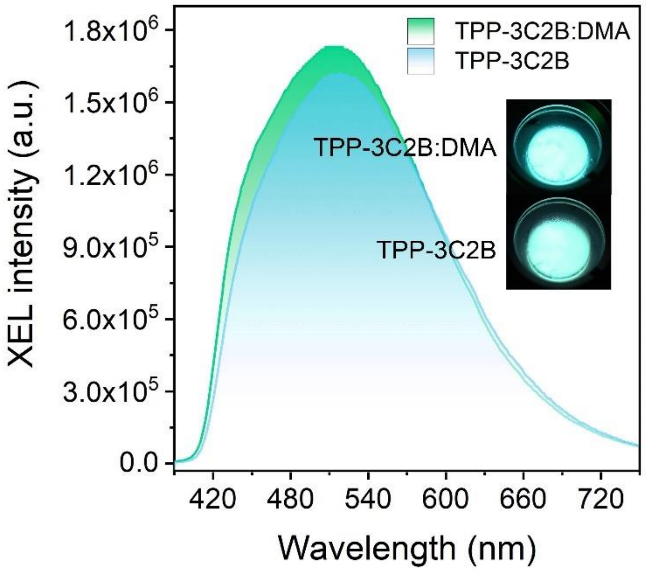

Supplementary Fig. 25 Comparison of the XEL spectra of TPP-3C2B and TPP-3C2B:DMA at 83 K.

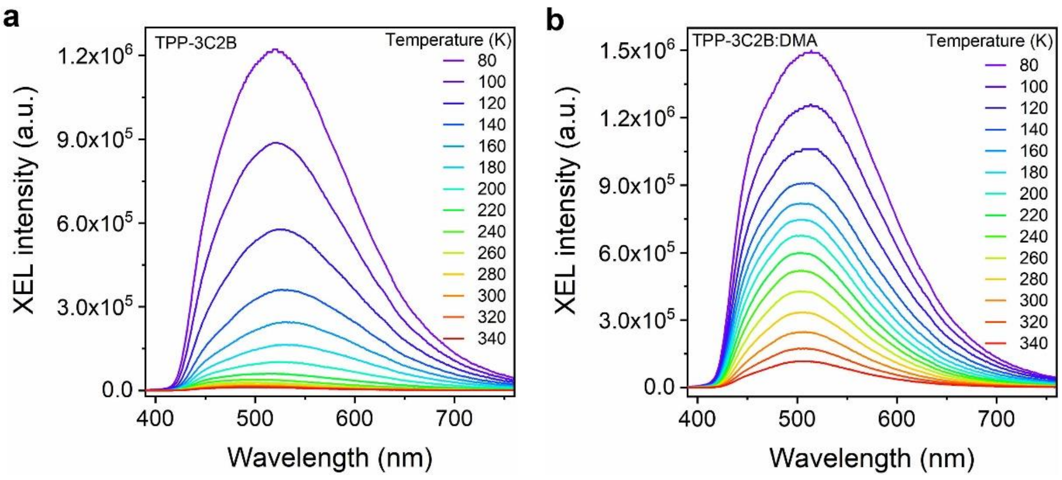

Supplementary Fig. 26 a-b, Temperature-dependent XEL spectra of **(a)** TPP-3C2B and **(b)** TPP-3C2B:DMA.

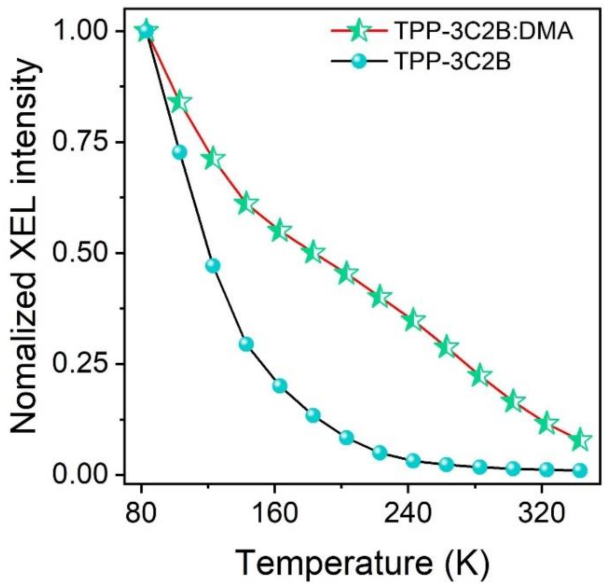

Supplementary Fig. 27 Temperature-dependent XEL intensity of TPP-3C2B and TPP-3C2B:DMA.

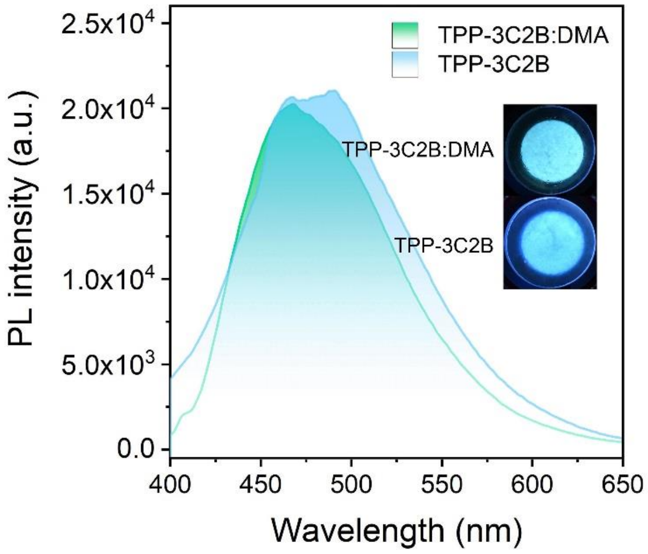

Supplementary Fig. 28 Comparison of the photoluminescence spectra of TPP-3C2B and TPP-3C2B:DMA at 83 K.

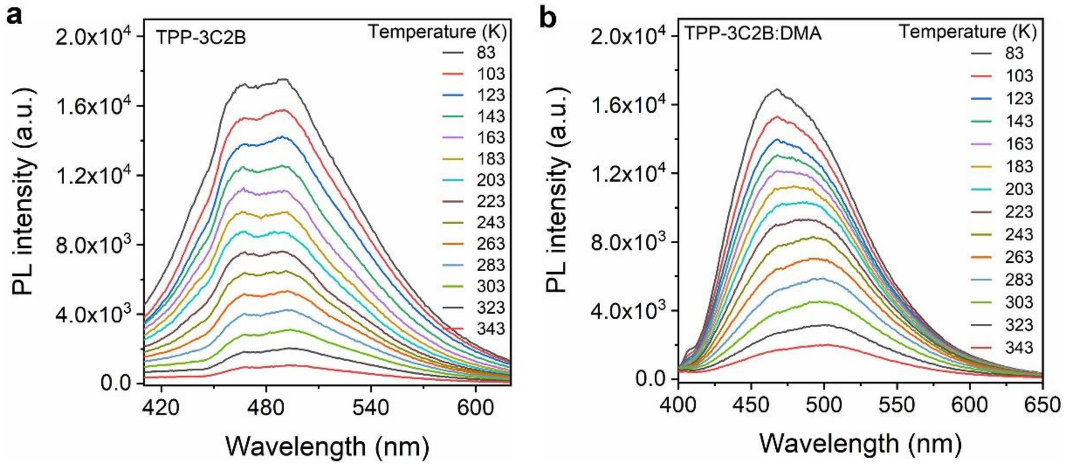

Supplementary Fig. 29 a-b, Temperature-dependent photoluminescence spectra of **(a)** TPP-3C2B and **(b)** TPP-3C2B:DMA.

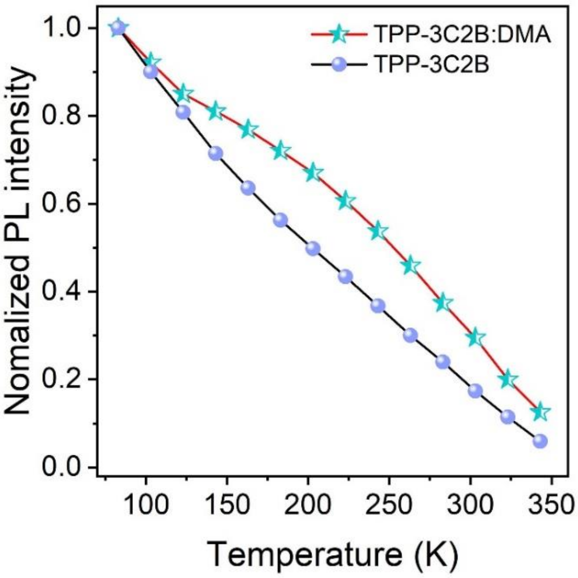

Supplementary Fig. 30 Temperature-dependent photoluminescence intensity of TPP-3C2B and TPP-3C2B:DMA.

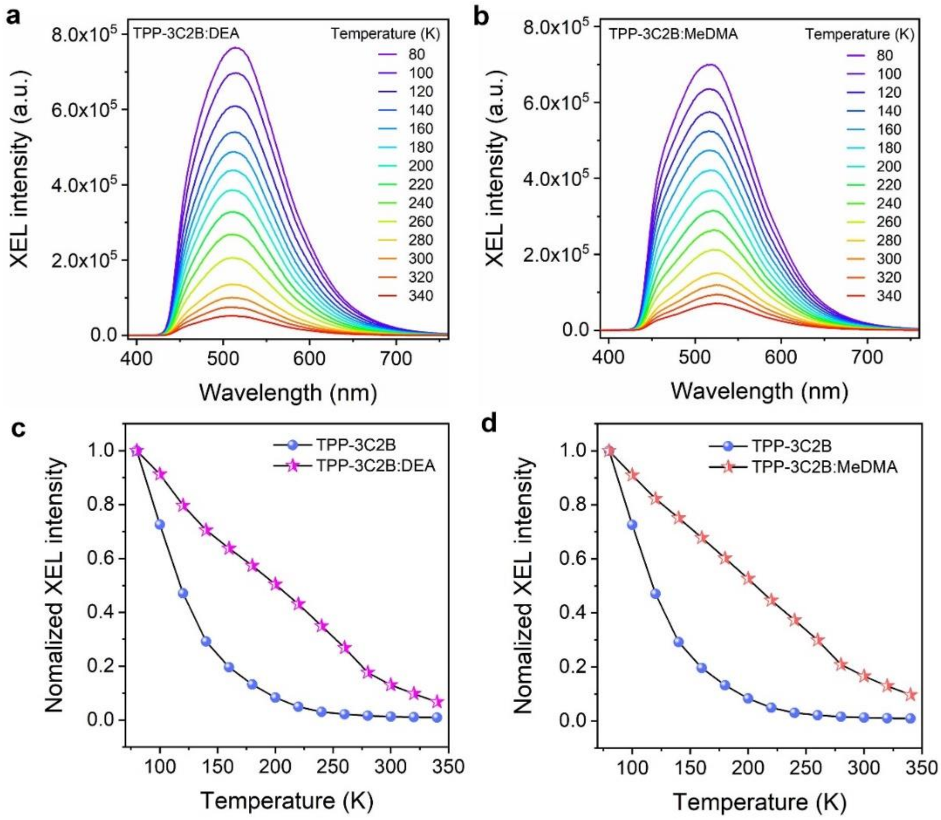

Supplementary Fig. 31 a-b, Temperature-dependent XEL spectra of **(a)** TPP-3C2B:DEA and **(b)** TPP-3C2B:MeDMA. **c,** Temperature-dependent XEL intensity of TPP-3C2B:DEA and TPP-3C2B. **d,** Temperature-dependent XEL intensity of TPP-3C2B:MeDMA and TPP-3C2B.

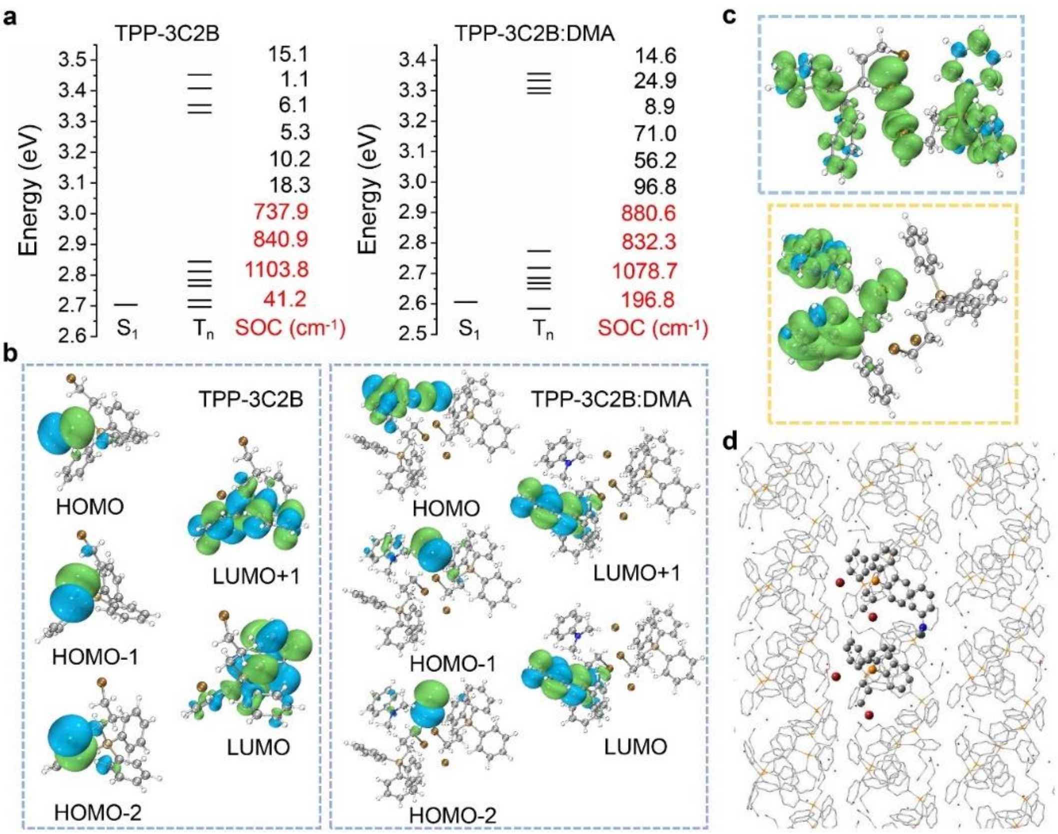

Supplementary Fig. 32 a, Energy level diagram and SOC constants. **b,** Electron density distributions of the highest occupied molecular orbitals (HOMOs) and lowest unoccupied molecular orbitals (LUMOs). **c,** Spin density localized on the ^{3}TPP-3C2B pair (up) and ^{3}TPP-3C2B:DMA moieties (down) (isovalue = 0.001). **d,** QM/MM model of TPP-3C2B:DMA.

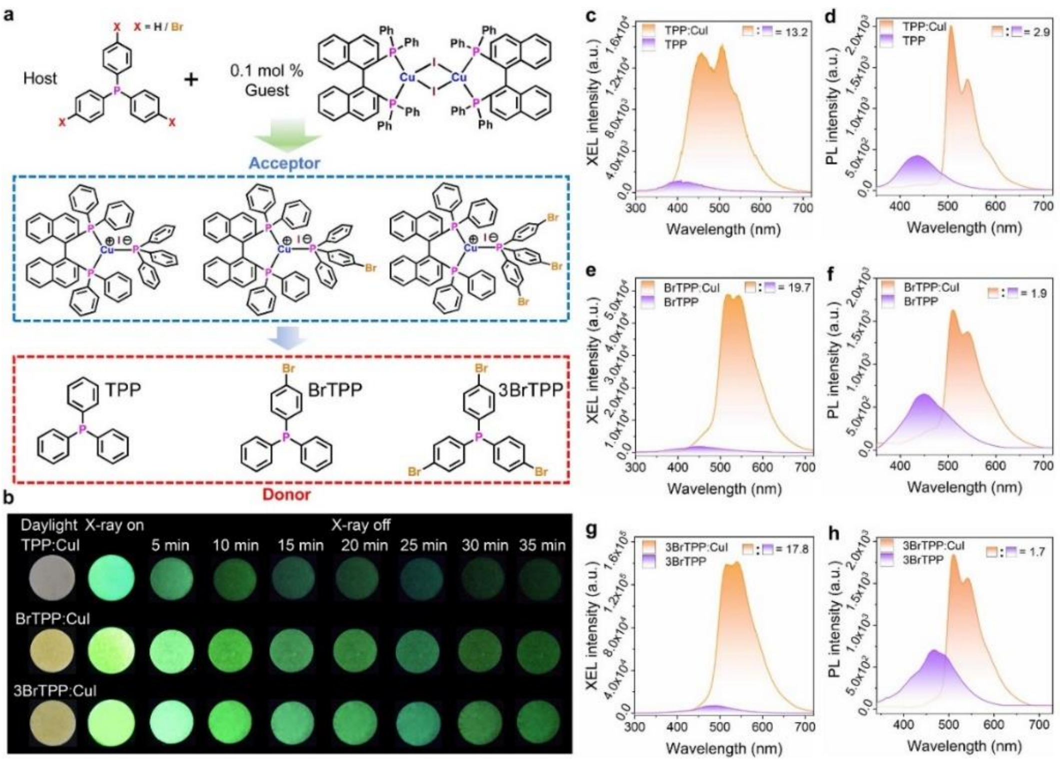

Supplementary Fig. 33 a, Synthesis of TPP:CuI, BrTPP:CuI and 3BrTPP:CuI. **b,** Photos of the X-ray excited afterglow of TPP:CuI, BrTPP:CuI and 3BrTPP:CuI. **c-h,** XEL spectra and integral area ratios of **(c)** TPP:CuI and TPP, **(e)** BrTPP:CuI and BrTPP, and **(g)** 3BrTPP:CuI and 3BrTPP under the same measurement conditions. Photoluminescence spectra and integral area ratios of **(d)** TPP:CuI and TPP, **(f)** BrTPP:CuI and BrTPP, and **(h)** 3BrTPP:CuI and 3BrTPP under the same measurement conditions.

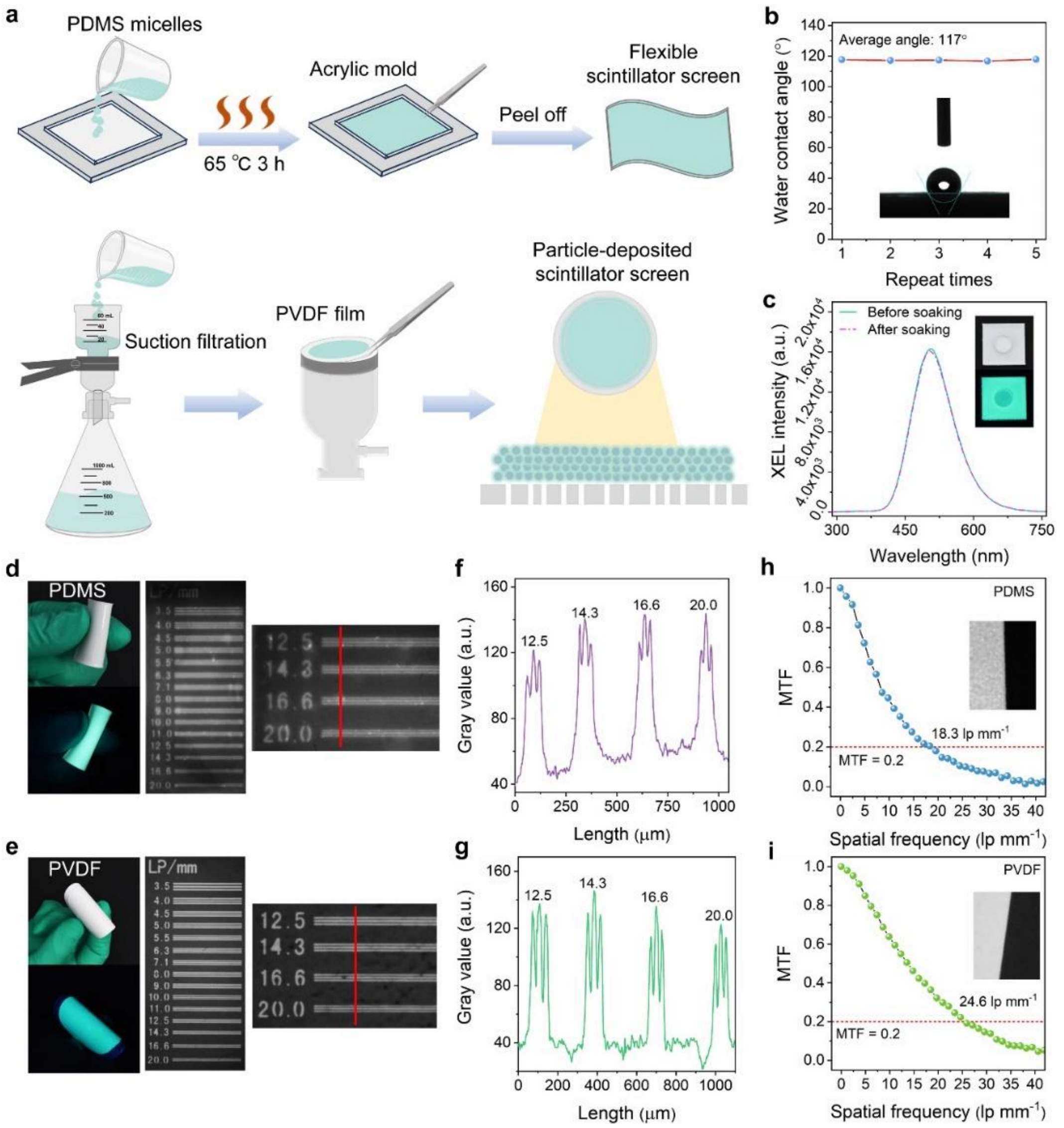

Supplementary Fig. 34 a, Preparation of the PDMS and PVDF scintillator screens. **b,** Interface contact angle between the scintillator screen and water droplet. **c,** XEL spectra of the scintillator screen before and after contact with water. Inset: photographs of the screen with water droplet under daylight and X-ray. **d,** Photo of the PDMS scintillator screen and imaging of the standard line-pair mask. **e,** Photo of the PVDF scintillator screen and imaging of the standard line-pair mask. **f-g,** Profile curves of the **(f)** PDMS scintillator screen and **(g)** PVDF scintillator screen plotted via ImageJ software along the red lines in **(d)** and **(e)**, respectively. **h-i,** MTF curves of the **(h)** PDMS scintillator screen and **(i)** PVDF scintillator screen. Inset: Photos of slanted-edge imaging.

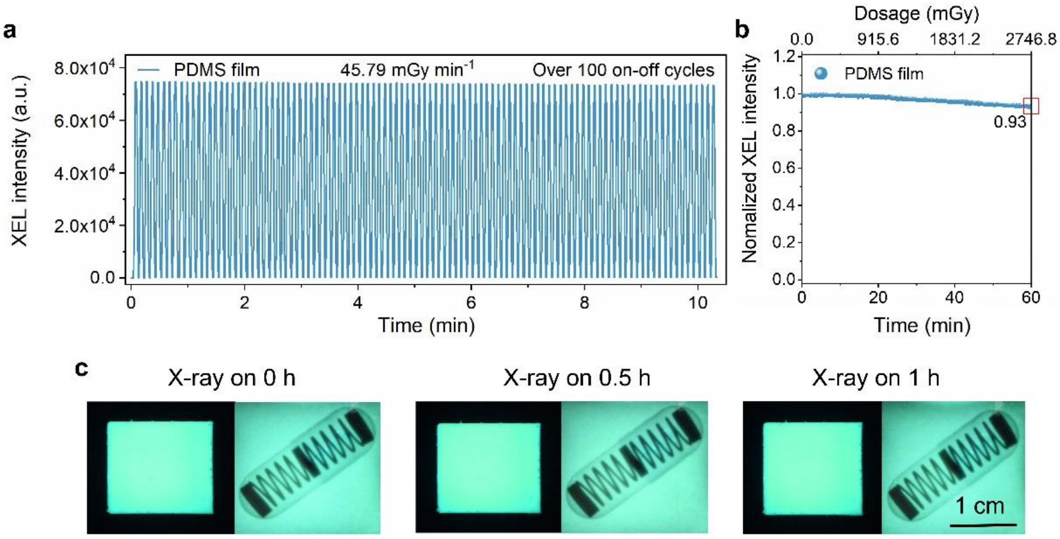

Supplementary Fig. 35 a, XEL intensities of the PDMS film of TPP-3C2B:DMA under X-ray irradiation (45.79 mGy min^{-1}) over 100 on-off cycles; each cycle lasted 3 s. **b,** XEL intensities of the PDMS film of TPP-3C2B:DMA under continuous X-ray irradiation for 60 min (2746.8 mGy in total). **c,** Photos of the PDMS film of TPP-3C2B:DMA and X-ray imaging of a spring using the films after continuous X-ray irradiation for different durations.

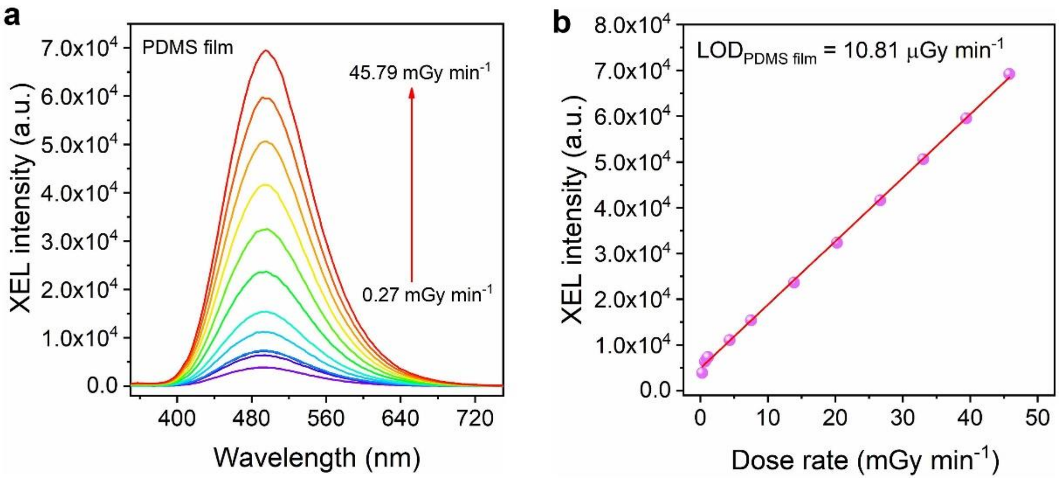

Supplementary Fig. 36 a, XEL spectra of the PDMS film of TPP-3C2B:DMA at different X-ray doses. **b,** Dose rate dependence of the XEL intensity of the PDMS film of TPP-3C2B:DMA.

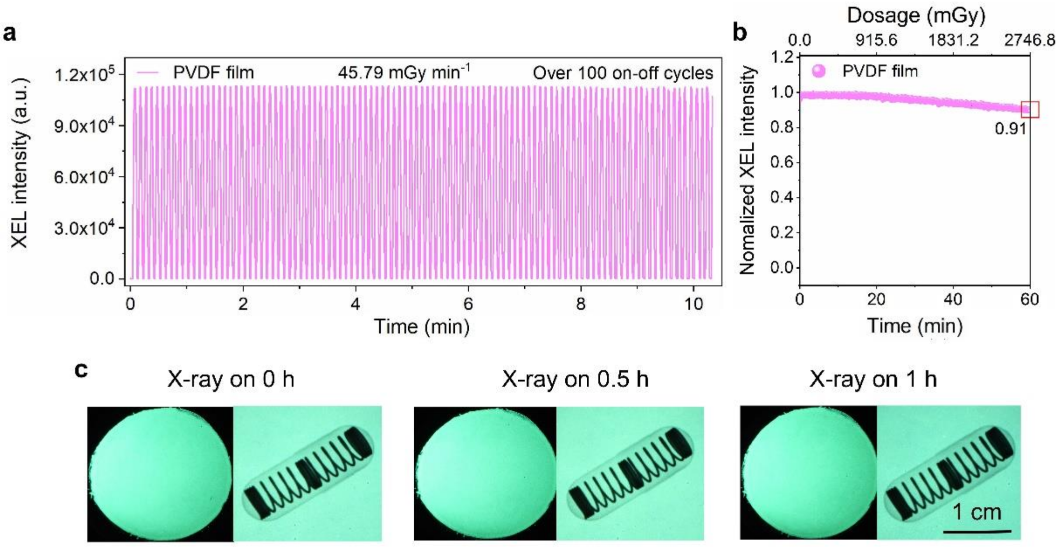

Supplementary Fig. 37 a, XEL intensities of the PVDF film of TPP-3C2B:DMA under X-ray irradiation (45.79 mGy min^{-1}) over 100 on-off cycles; each cycle lasted 3 s. **b,** XEL intensities of the PVDF film of TPP-3C2B:DMA under continuous X-ray irradiation for 60 min (2746.8 mGy in total). **c,** Photos of the PVDF film of TPP-3C2B:DMA and X-ray imaging of a spring using the films after continuous X-ray irradiation for different durations.

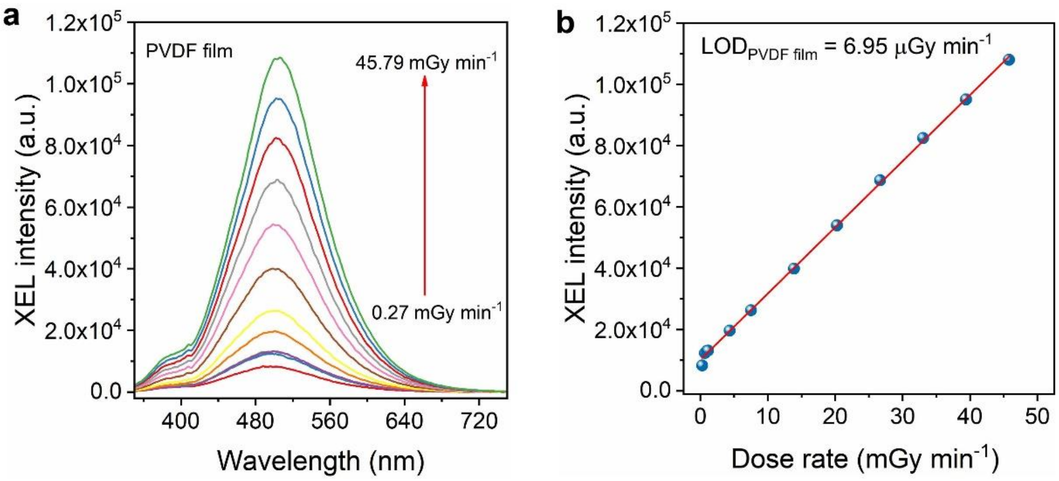

Supplementary Fig. 38 a, XEL spectra of the PVDF film of TPP-3C2B:DMA at different X-ray doses. **b,** Dose rate dependence of the XEL intensity of the PVDF film of TPP-3C2B:DMA.

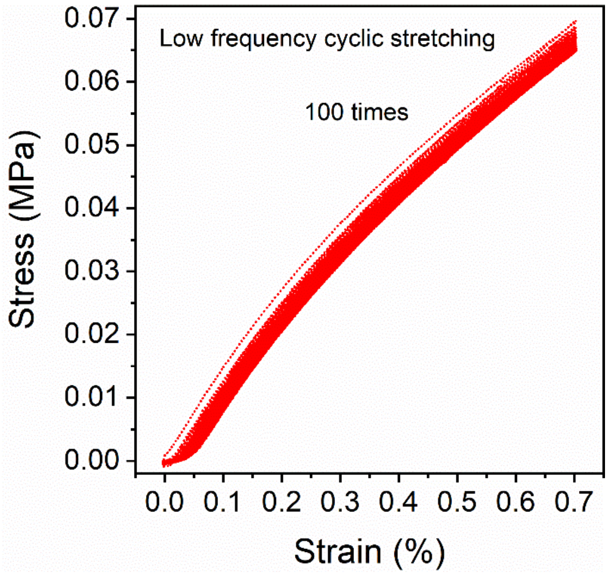

Supplementary Fig. 39 Stress-strain curve corresponding to low-frequency cyclic stretching (clamping distance = 15 mm, tensile rate = 20 mm min^{-1}, tensile strength = 0.7 MPa).

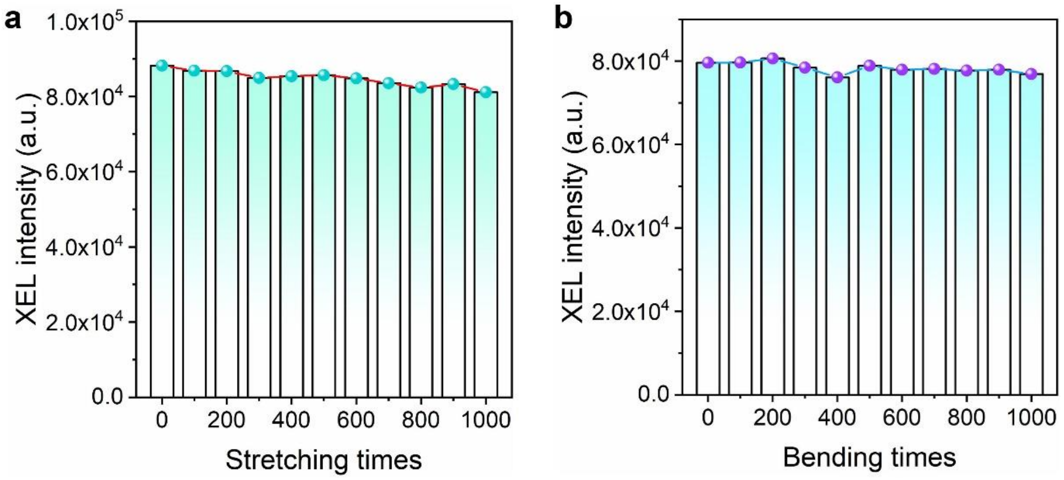

Supplementary Fig. 40 a-b, XEL intensity of the scintillator screen at 510 nm after **(a)** stretching and **(b)** bending for different times.

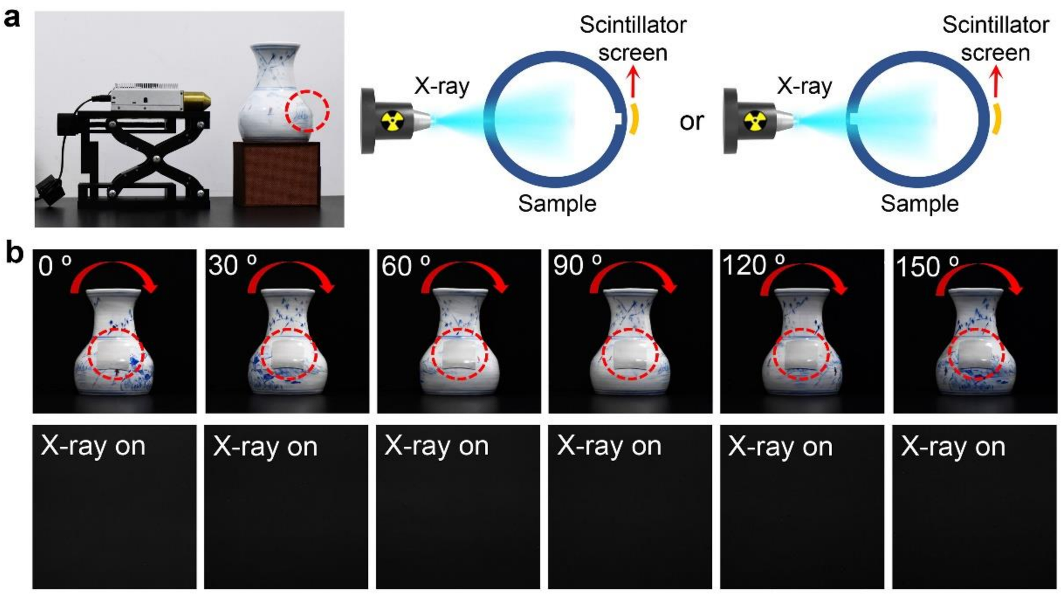

Supplementary Fig. 41 a, Schematic diagram of direct X-ray imaging. **b,** Direct X-ray imaging of the bottle.

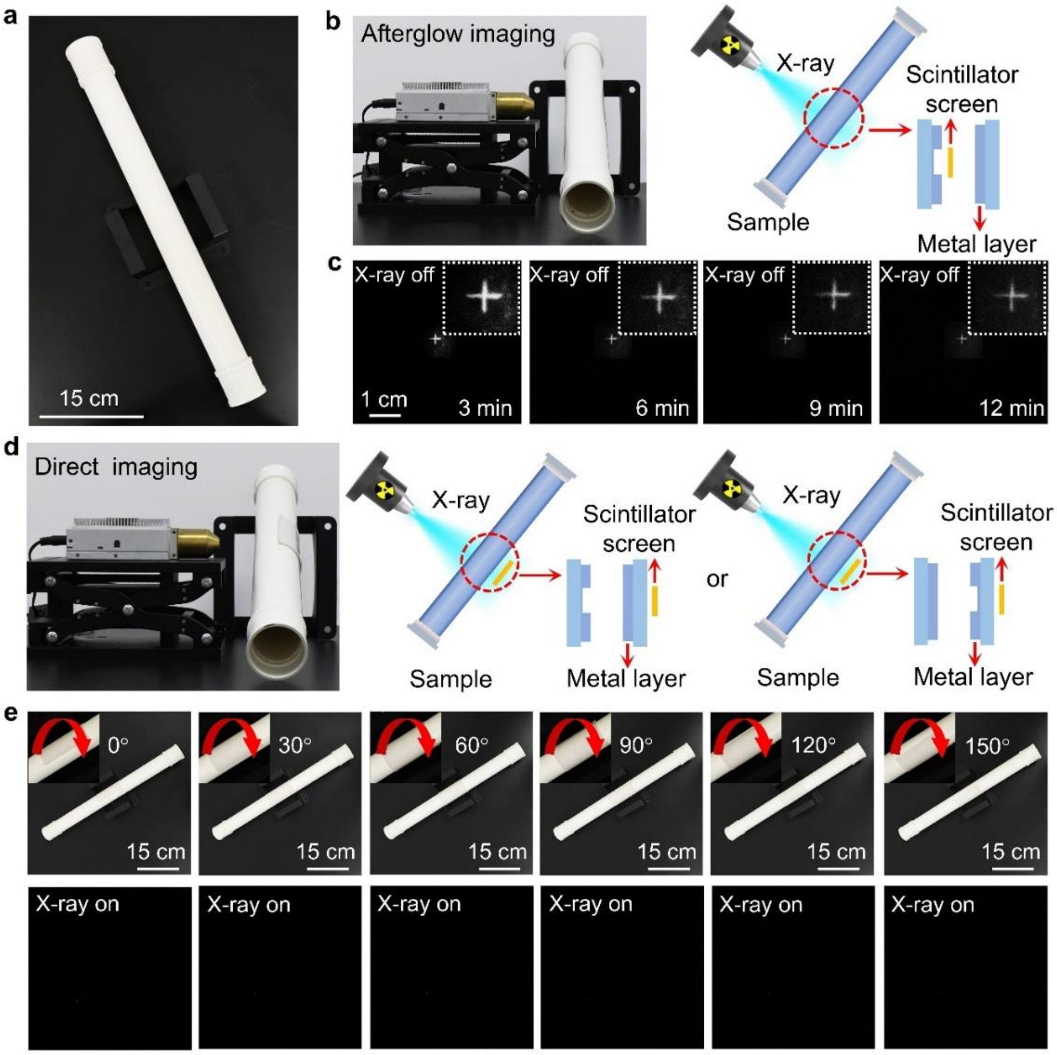

Supplementary Fig. 42 a, Photo of a metal-plastic composite pipe. **b,** Schematic diagram of X-ray afterglow imaging. **c,** X-ray afterglow imaging of a cross-shaped scratch inside the pipe. **d,** Schematic diagram of direct X-ray imaging. **e,** Direct X-ray imaging of the pipe.

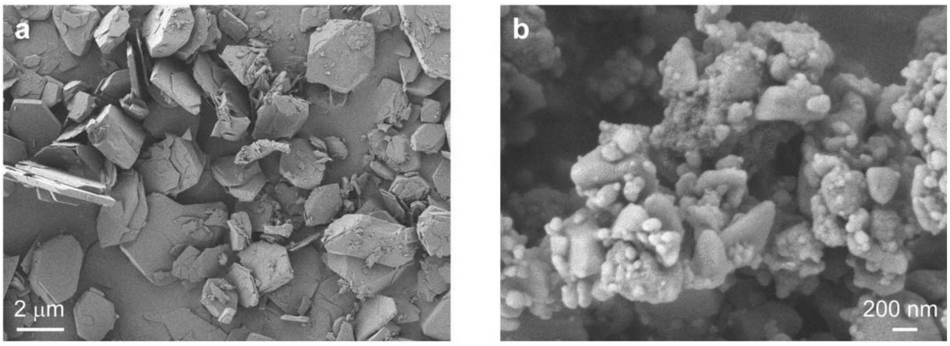

Supplementary Fig. 43 a-b, SEM images of **(a)** microcrystals and **(b)** nanocrystals.

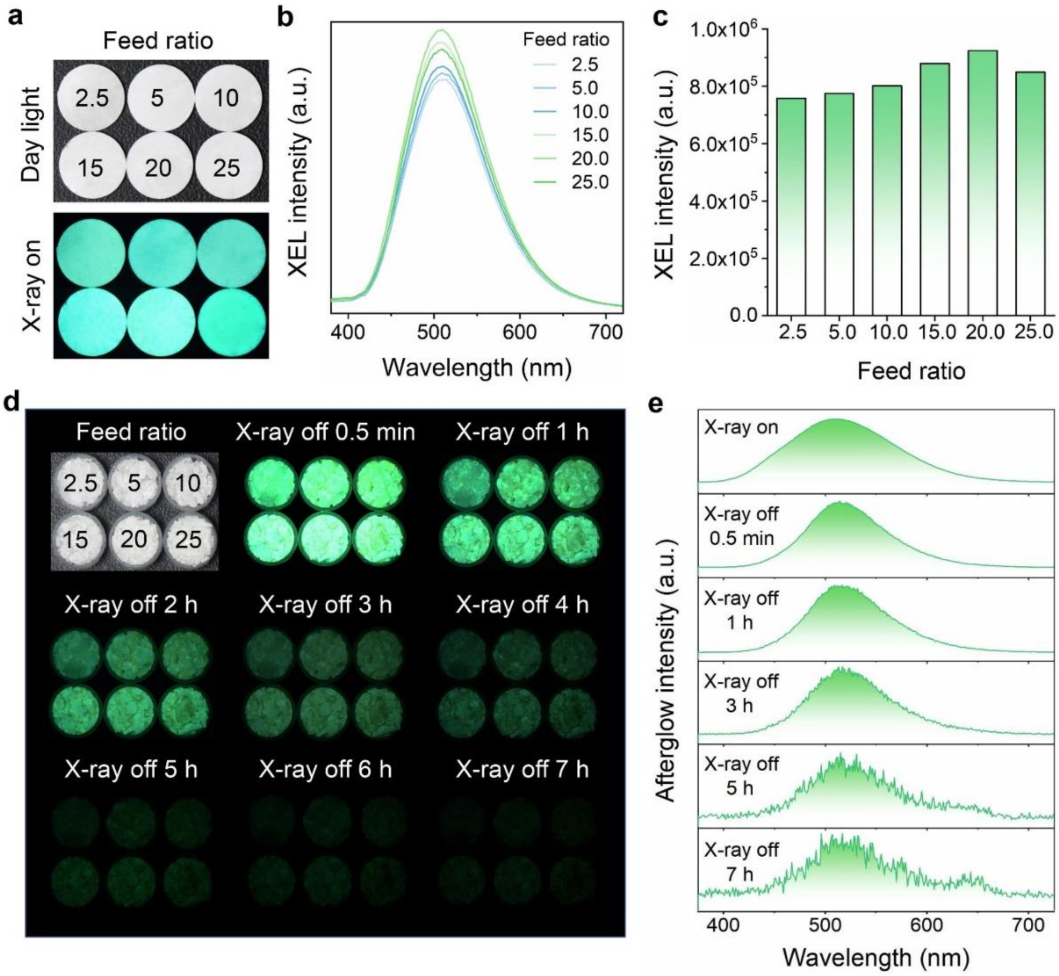

Supplementary Fig. 44 a, Photos of TPP-3C2B:DMA wafers synthesized with different DMA feeding ratios. **b-c,** (b) XEL spectra and (c) XEL intensities at 510 nm of TPP-3C2B:DMA wafers. **d,** Afterglow photographs of TPP-3C2B:DMA wafers. **e,** X-ray excited afterglow spectra of TPP-3C2B:DMA (DMA feeding ratio of 20).

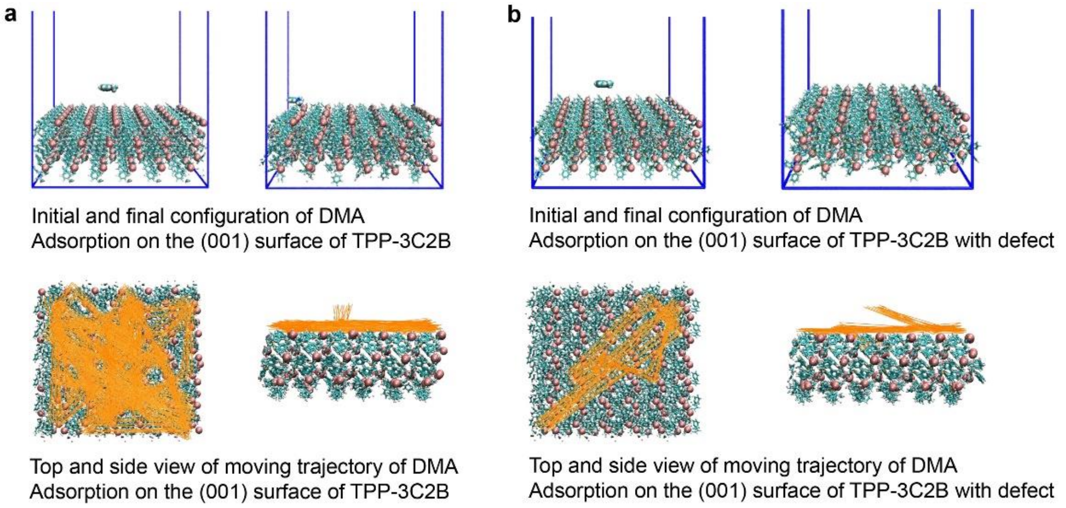

Supplementary Fig. 45 a-b, DMA molecule integrated into **(a)** the flawless TPP-3C2B surface and **(b)** the TPP-3C2B surface with a defect after a 50 ns MD simulation.

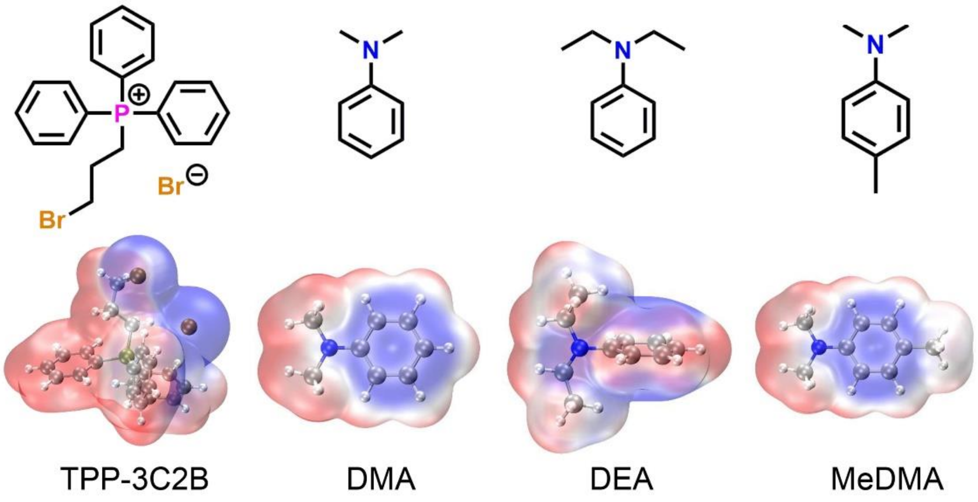

	TPP-3C2B	DMA	DEA	MeDMA
V_{vdW} (Å^3)	472.909	174.948	224.550	200.316
x (Å)	9.877	9.506	9.439	10.440
y (Å)	11.650	6.733	9.084	6.729
z (Å)	11.656	4.183	6.797	4.380

Supplementary Fig. 46 Electrostatic potential (ESP) surfaces, van der Waals volumes, and molecular sizes of TPP-3C2B, DMA, DEA and MeDMA.

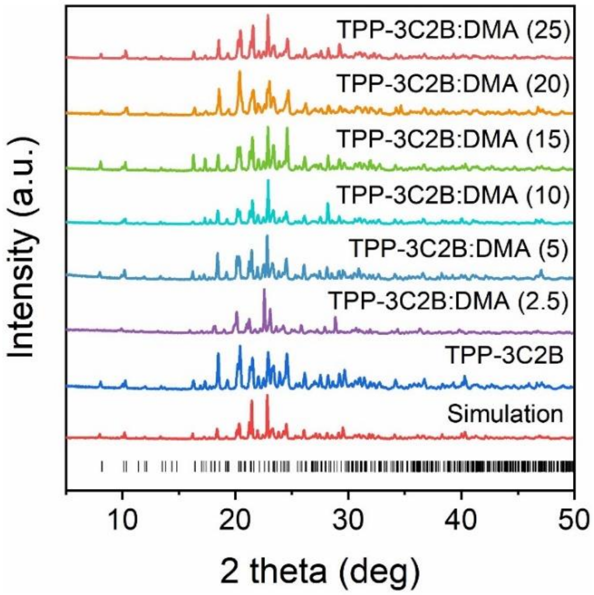

Supplementary Fig. 47 PXRD data of TPP-3C2B:DMA with different DMA feeding ratios.

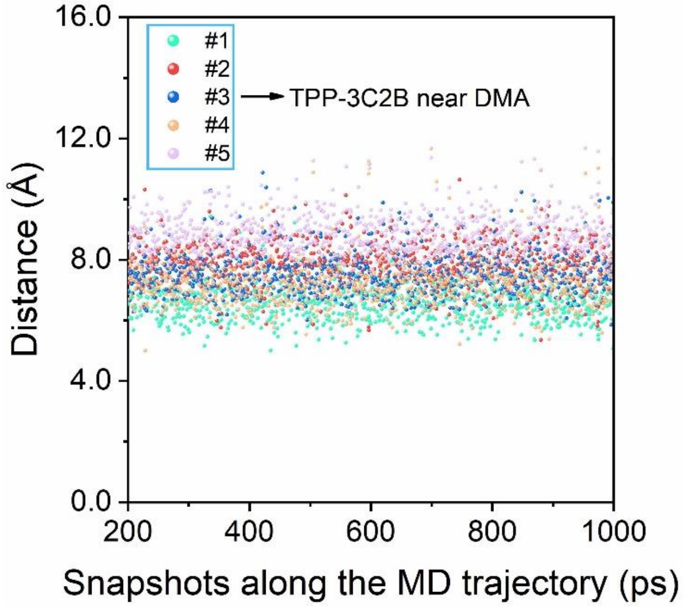

Supplementary Fig. 48 Distance between DMA and TPP-3C2B during MD simulation.

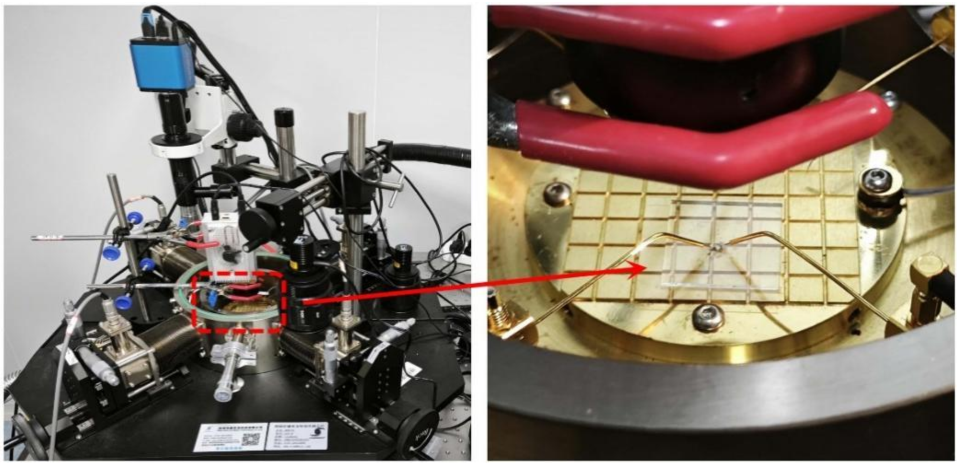

Supplementary Fig. 49 Device for photoconductive gain measurements.

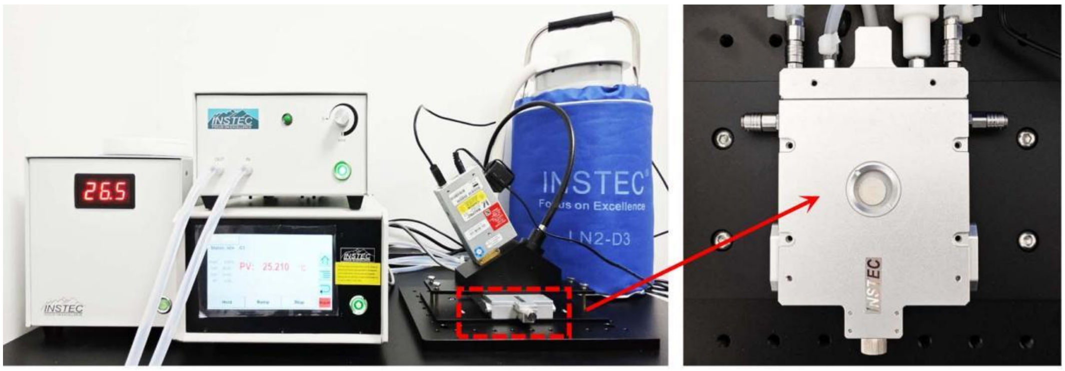

Supplementary Fig. 50 Device for temperature-dependent radioluminescence measurements.

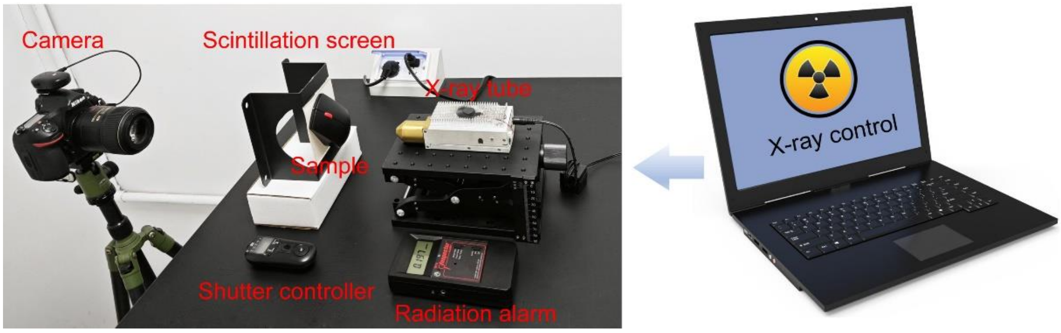

Supplementary Fig. 51 Schematic diagram of the X-ray imaging device.

Supplementary Table 1. Crystal data and structure refinements of TPP-3C2B.

	TPP-3C2B
Empirical formula	$C_{21}H_{21}Br_2P$
Formula weight	464.17
Temperature (K)	200
Crystal system	monoclinic
Space group	$P2_1/c$
a (Å)	11.0908(2)
b (Å)	10.1179(2)
c (Å)	17.5478(3)
α (°)	90
β (°)	104.846(6)
γ (°)	90
Volume (Å^3)	1903.40(6)
Z	4
ρ_{calc} (g cm^{-3})	1.620
μ (mm^{-1})	6.175
F(000)	928.0
Crystal size (mm^3)	0.4×0.3×0.15
Radiation	Cu Kα (λ = 1.54184 Å)
2θ range for data collection (°)	8.248 to 149.986
Index ranges	$-13 \leq h \leq 12$, $-10 \leq k \leq 12$, $-19 \leq l \leq 21$
Reflections collected	10335
Independent reflections	3727 [R_{int} = 0.0308, R_{sigma} = 0.0219]
Data/restraints/parameters	3727/30/218
Goodness-of-fit on F^2	1.056
Final R indexes [I>=2σ (I)]	R_1 = 0.0385, wR_2 = 0.1051
Final R indexes [all data]	R_1 = 0.0392, wR_2 = 0.0157
CCDC	2373173

Supplementary Table 2. Crystal data and structure refinements of TPP-3C2B:DMA(2.5) and TPP-3C2B:DMA(5).

	TPP-3C2B-DMA(2.5)	TPP-3C2B-DMA(5)
Empirical formula	$C_{21}H_{21}Br_2P$	$C_{21}H_{21}Br_2P$
Formula weight	464.17	464.17
Temperature (K)	200	200
Crystal system	monoclinic	monoclinic
Space group	$P2_1/c$	$P2_1/c$
a (Å)	11.1044(3)	11.0965(2)
b (Å)	10.1266(3)	10.12890(10)
c (Å)	17.5319(5)	17.5393(2)
α (°)	90	90
β (°)	104.849(3)	104.860(2)
γ (°)	90	90
Volume (Å^3)	1905.62(10)	1905.40(5)
Z	4	4
ρ_{calc} (g cm^{-3})	1.618	1.618
μ (mm^{-1})	6.167	6.168
F(000)	928.0	928.0
Crystal size (mm^3)	0.4×0.3×0.25	0.35×0.2×0.15
Radiation	Cu Kα (λ = 1.54184 Å)	Cu Kα (λ = 1.54184 Å)
2θrange for data collection (°)	8.238 to 149.264	10.174 to 150.696
Index ranges	$-13 \le h \le 13$, $-12 \le k \le 12$, $-17 \le l \le 21$	$-12 \le h \le 13$, $-12 \le k \le 12$, $-22 \le l \le 21$
Reflections collected	12133	10398
Independent reflections	3736 [R_{int} = 0.0253, R_{sigma} = 0.0194]	3753 [R_{int} = 0.0368, R_{sigma} = 0.0266]
Data/restraints/parameters	3736/0/217	3753/0/208
Goodness-of-fit on F^2	1.079	1.089
Final R indexes [I>=2σ (I)]	R_1 = 0.0314, wR_2 = 0.0875	R_1 = 0.0505, wR_2 = 0.1421
Final R indexes [all data]	R_1 = 0.0330, wR_2 = 0.0886	R_1 = 0.0510, wR_2 = 0.1427
CCDC	2373167	2373166

Supplementary Table 3. Crystal data and structure refinements of TPP-3C2B:DMA(10) and TPP-3C2B:DMA(15).

	TPP-3C2B-DMA(10)	TPP-3C2B-DMA(15)
Empirical formula	$C_{21}H_{21}Br_2P$	$C_{21}H_{21}Br_2P$
Formula weight	464.17	464.17
Temperature (K)	200	200
Crystal system	monoclinic	monoclinic
Space group	$P2_1/c$	$P2_1/c$
a (Å)	11.1004(2)	11.0979(2)
b (Å)	10.1265(2)	10.12930(10)
c (Å)	17.5401(3)	17.5379(2)
α (°)	90	90
β (°)	104.834(2)	104.8850(10)
γ (°)	90	90
Volume (Å^3)	1905.94(6)	1905.35 (5)
Z	4	4
ρ_{calc} (g cm^{-3})	1.618	1.618
μ (mm^{-1})	6.166	6.168
F(000)	928.0	928.0
Crystal size (mm^3)	0.25×0.2×0.12	0.5×0.4×0.2
Radiation	Cu Kα (λ = 1.54184 Å)	Cu Kα (λ = 1.54184 Å)
2θ range for data collection (°)	8.24 to 149.834	8.244 to 150.57
Index ranges	$-13 \leq h \leq 13$, $-12 \leq k \leq 12$, $-21 \leq l \leq 19$	$-13 \leq h \leq 13$, $-12 \leq k \leq 10$, $-21 \leq l \leq 21$
Reflections collected	11104	10796
Independent reflections	3751 [R_{int} = 0.0256, R_{sigma} = 0.0186]	3758 [R_{int} = 0.0208, R_{sigma} = 0.0147]
Data/restraints/parameters	3751/0/217	3758/0/217
Goodness-of-fit on F^2	1.048	1.089
Final R indexes [I>=2σ (I)]	R_1 = 0.0338, wR_2 = 0.0930	R_1 = 0.0345, wR_2 = 0.0907
Final R indexes [all data]	R_1 = 0.0345, wR_2 = 0.0936	R_1 = 0.0349, wR_2 = 0.0910
CCDC	2373165	2373169

Supplementary Table 4. Crystal data and structure refinements of TPP-3C2B:DMA(20) and TPP-3C2B:DMA(25).

	TPP-3C2B-DMA(20)	TPP-3C2B-DMA(25)
Empirical formula	$C_{21}H_{21}Br_2P$	$C_{21}H_{21}Br_2P$
Formula weight	464.17	464.17
Temperature (K)	200	200
Crystal system	monoclinic	monoclinic
Space group	$P2_1/c$	$P2_1/c$
a (Å)	11.0970(3)	11.0979(2)
b (Å)	10.1207(3)	10.1269(2)
c (Å)	17.5511(5)	17.5362(3)
α (°)	90	90
β (°)	104.823(3)	104.843(2)
γ (°)	90	90
Volume (Å^3)	1905.55(10)	1905.08 (6)
Z	4	4
ρ_{calc} (g cm^{-3})	1.618	1.618
μ (mm^{-1})	6.168	6.169
F(000)	928.0	928.0
Crystal size (mm^3)	0.4×0.2×0.18	0.5×0.45×0.3
Radiation	Cu Kα (λ = 1.54184 Å)	Cu Kα (λ = 1.54184 Å)
2θ range for data collection (°)	8.242 to 149.914	8.244 to 150.54
Index ranges	$-8 \le h \le 13$, $-10 \le k \le 12$, $-21 \le l \le 21$	$-13 \le h \le 13$, $-9 \le k \le 12$, $-20 \le l \le 21$
Reflections collected	10364	9734
Independent reflections	3724 [R_{int} = 0.0304, R_{sigma} = 0.0250]	3765 [R_{int} = 0.0369, R_{sigma} = 0.0261]
Data/restraints/parameters	3724/0/218	3765/0/218
Goodness-of-fit on F^2	1.071	1.138
Final R indexes [I>=2σ (I)]	R_1 = 0.0388, wR_2 = 0.1105	R_1 = 0.0480, wR_2 = 0.1323
Final R indexes [all data]	R_1 = 0.0404, wR_2 = 0.1140	R_1 = 0.0484, wR_2 = 0.1329
CCDC	2373175	2373174

Supplementary Table 5. Trap depth (in eV) obtained by fitting of afterglow curves

and Hoogenstraaten method.

	Fitting of afterglow curves	Hoogenstraaten method
TPP-3C2B:DMA	0.370	0.557
TPP-3C2B:DEA	0.336	0.425
TPP-3C2B:MeDEA	0.307	0.357

Supplementary Table 6. The spin-orbit coupling matrix element of the lowest 10 singlet and triplet excited states of TPP-3C2B at the PBE0/def2-SVP theoretical level (in cm^{-1}).

	S_1	S_2	S_3	S_4	S_5	S_6	S_7	S_8	S_9	S_{10}
T_1	41.2	1098.3	1043.2	381.1	117.6	128.2	5.4	8.7	3.4	14.0
T_2	1103.8	65.8	1054.7	285.9	113.3	109.2	2.5	3.9	4.7	11.0
T_3	840.8	836.5	164.5	115.2	700.0	710.6	8.3	11.1	6.8	45.1
T_4	737.9	718.7	111.8	139.0	827.5	829.2	4.3	3.2	3.5	33.1
T_5	18.3	110.1	318.6	1033.8	413.8	1034.5	2.3	5.9	6.2	25.6
T_6	10.2	42.5	319.2	1065.7	1030.0	383.7	1.9	4.8	4.8	25.2
T_7	5.3	9.3	4.6	14.1	10.4	11.0	26.8	1107.1	1107.5	15.6
T_8	1.1	5.4	4.6	6.6	5.9	8.8	1105.6	37.1	1093.9	30.5
T_9	6.1	9.1	4.3	8.0	11.5	11.5	1105.5	1093.2	33.0	17.1
T_{10}	15.1	8.1	17.9	23.5	33.9	37.7	104.8	90.4	78.5	148.4

Supplementary Table 7. The spin-orbit coupling matrix element of the lowest 10 singlet and triplet excited states of TPP-3C2B:DMA at the PBE0/def2-SVP theoretical level (in cm^{-1}).

	S_1	S_2	S_3	S_4	S_5	S_6	S_7	S_8	S_9	S_{10}
T_1	196.8	1151.3	78.5	1125.5	142.9	110.2	57.0	0.9	23.0	41.1
T_2	1078.7	512.0	113.5	973.2	357.1	347.2	23.6	0.8	3.3	35.7
T_3	832.3	582.2	102.2	577.4	798.3	820.2	20.6	2.8	6.1	23.4
T_4	880.6	828.2	31.2	199.3	779.1	740.1	10.6	2.5	3.9	19.1
T_5	96.8	65.1	1151.4	160.1	49.5	1135.6	5.0	0.8	5.1	3.4
T_6	56.2	167.1	1157.9	135.5	1121.1	51.9	18.8	3.7	9.5	24.5
T_7	71.0	45.8	6.4	23.8	15.2	1.8	19.0	4.3	79.1	1145.0
T_8	8.9	11.1	6.3	10.9	2.7	5.9	29.5	0.1	28.2	49.0
T_9	24.9	32.9	12.3	25.8	18.8	14.4	100.5	1.2	134.0	58.1
T_{10}	14.6	7.5	2.7	11.9	5.9	8.2	519.8	2.1	53.8	100.0

Supplementary Table 8. The energy levels (in eV) of S_n and T_n for TPP-3C2B and TPP-3C2B:DMA, respectively.

	TPP-3C2B	TPP-3C2B:DMA		TPP-3C2B	TPP-3C2B:DMA
S_1	2.703	2.606	T_1	2.696	2.584
S_2	2.725	2.669	T_2	2.718	2.650
S_3	2.776	2.679	T_3	2.765	2.668
S_4	2.794	2.707	T_4	2.783	2.685
S_5	2.835	2.724	T_5	2.812	2.718
S_6	2.862	2.779	T_6	2.844	2.773
S_7	3.331	3.302	T_7	3.329	3.292
S_8	3.356	3.324	T_8	3.354	3.308
S_9	3.410	3.350	T_9	3.408	3.334
S_{10}	3.482	3.365	T_{10}	3.452	3.356

Supplementary Table 9. Mulliken Net Atomic Charges and Spin for Triplet Localized on TPP-3C2B.

Atom	Charge (a.u.)	Spin (a.u.)
1 Br	-0.60019	0.013085
2 Br	-0.113177	0.000527
3 P	0.525362	0.077745
4 C	-0.085116	0.00136
5 H	0.092518	0.000009
6 H	0.09028	0.001739
7 C	-0.043226	0.00275
8 H	0.054558	-0.000168
9 H	0.074343	-0.000158
10 C	-0.181339	-0.003802
11 H	0.118707	-0.000339
12 H	0.093202	-0.002731
13 C	-0.225542	0.06534
14 C	-0.013478	0.059693
15 H	0.025955	-0.003678
16 C	-0.018117	-0.035667
17 H	0.023199	0.000761
18 C	-0.032673	0.16769
19 H	0.022627	-0.009009
20 C	-0.014131	-0.058448
21 H	0.024928	0.00188
22 C	-0.012507	0.094471
23 H	0.032167	-0.005233
24 C	-0.192905	0.00006
25 C	-0.017031	0.044112
26 H	0.050275	-0.00239
27 C	-0.012283	-0.02228
28 H	0.034691	0.000931
29 C	-0.008909	0.044533
30 H	0.028729	-0.002411
31 C	-0.021298	-0.000049

32 H	0.025806	-0.000272
33 C	-0.000356	0.008554
34 H	0.034408	-0.000641
35 C	-0.157031	0.018574
36 C	0.006446	0.012572
37 H	0.041829	-0.001096
38 C	-0.025918	-0.007277
39 H	0.02755	0.000441
40 C	-0.016804	0.016038
41 H	0.032787	-0.000867
42 C	-0.020081	-0.001053
43 H	0.037962	0.000339
44 C	0.046904	0.004444
45 H	0.035783	-0.000082
46 Br	-0.211292	0.721907
47 Br	-0.009005	0.259497
48 P	0.487646	0.130422
49 C	-0.100916	-0.002763
50 H	0.110863	0.008991
51 H	0.126936	0.002707
52 C	-0.023217	0.001169
53 H	0.089732	-0.000414
54 H	0.063204	-0.000287
55 C	-0.218956	-0.000324
56 H	0.092322	-0.001903
57 H	0.139988	0.002762
58 C	-0.162277	0.005806
59 C	0.000438	0.057736
60 H	0.036701	-0.003366
61 C	-0.014736	-0.035422
62 H	0.039353	0.001373
63 C	-0.015501	0.095747
64 H	0.018764	-0.005076
65 C	-0.016121	-0.037902

66 H	0.020265	0.00141
67 C	-0.026434	0.058316
68 H	0.023948	-0.002648
69 C	-0.195616	0.012719
70 C	0.000467	0.050196
71 H	0.045243	-0.002735
72 C	-0.012605	-0.031425
73 H	0.029497	0.001203
74 C	-0.017445	0.083763
75 H	0.028035	-0.0044
76 C	-0.017737	-0.019494
77 H	0.027829	0.000594
78 C	-0.013495	0.032003
79 H	0.045336	-0.001932
80 C	-0.156295	0.02013
81 C	-0.011746	0.052917
82 H	0.041754	-0.002915
83 C	-0.012155	-0.033396
84 H	0.025366	0.001503
85 C	-0.016676	0.092398
86 H	0.024087	-0.004887
87 C	-0.01542	-0.031833
88 H	0.022616	0.001229
89 C	-0.011437	0.049289
90 H	0.02607	-0.001895
91 C	-0.092406	0.000052
92 C	0.195905	0.000073
93 C	-0.076415	-0.000045
94 C	-0.046607	-0.000165
95 C	-0.021442	0.000065
96 C	-0.023424	-0.000071
97 H	-0.006873	-0.000007
98 H	0.013949	-0.000529
99 H	0.00342	0.00039

100 H	0.000128	-0.000001
101 H	0.00903	0.000003
102 N	-0.406116	-0.00006
103 C	0.067536	-0.000037
104 H	0.042476	0.000001
105 H	0.03663	0.000008
106 H	0.049887	0.000019
107 C	0.053106	0.000032
108 H	0.034084	0.000002
109 H	0.081918	-0.00054
110 H	0.028935	0.000042

Supplementary Table 10. Mulliken Net Atomic Charges and Spin for Triplet Localized on DMA and TPP-3C2B.

Atom	Charge (a.u.)	Spin (a.u.)
1 Br	-0.480736	0.283556
2 Br	-0.172277	-0.000024
3 P	0.523376	-0.000247
4 C	-0.083778	0.000003
5 H	0.088169	0.000002
6 H	0.126097	0.000041
7 C	-0.031957	-0.000018
8 H	0.064638	0.000003
9 H	0.058053	0
10 C	-0.172655	0.000309
11 H	0.111006	-0.001619
12 H	0.113825	0.000001
13 C	-0.163629	0.00003
14 C	0.012241	0.000027
15 H	0.042323	-0.000015
16 C	-0.014095	-0.000008
17 H	0.044936	0.000004
18 C	-0.001735	0.000017
19 H	0.035478	-0.000001
20 C	-0.016573	-0.000011
21 H	0.031308	0
22 C	-0.01535	0.000027
23 H	0.031649	0.000008
24 C	-0.20822	0.000022
25 C	0.01593	0.000653
26 H	0.055992	-0.000681
27 C	-0.013342	-0.000054
28 H	0.039872	0.000003
29 C	-0.001003	0.000051
30 H	0.037224	0.000005
31 C	-0.014768	-0.000046

32 H	0.034134	0.000004
33 C	-0.006253	0.000056
34 H	0.042867	0
35 C	-0.12646	0.002074
36 C	0.021057	0.001066
37 H	0.059474	-0.000074
38 C	-0.011688	-0.00017
39 H	0.037543	0.000011
40 C	0.005632	0.000263
41 H	0.030629	-0.000016
42 C	-0.011488	-0.000238
43 H	0.026411	0.000008
44 C	-0.004416	0.00057
45 H	0.032878	-0.000029
46 Br	-0.637201	0.000925
47 Br	-0.141754	0.000226
48 P	0.480073	0.145314
49 C	-0.077742	0.000702
50 H	0.083701	0.000692
51 H	0.107478	-0.000968
52 C	-0.07556	-0.001341
53 H	0.074467	-0.001282
54 H	0.070484	0.001192
55 C	-0.146852	0.024938
56 H	0.112658	0.005559
57 H	0.071112	0.000533
58 C	-0.265044	0.19325
59 C	-0.059175	0.277573
60 H	-0.001605	-0.014655
61 C	-0.030568	-0.165501
62 H	-0.00168	0.005569
63 C	-0.09143	0.469373
64 H	-0.006678	-0.024843
65 C	-0.041684	-0.077227

66 H	-0.001303	0.001159
67 C	-0.022909	0.156258
68 H	0.01584	-0.009139
69 C	-0.225168	0.013664
70 C	0.017867	0.003465
71 H	0.051425	0.000161
72 C	-0.022789	0.003615
73 H	0.030713	0.000584
74 C	-0.005669	-0.001172
75 H	0.024201	0.000012
76 C	-0.021435	0.006823
77 H	0.02281	-0.000276
78 C	0.002683	-0.000358
79 H	0.051962	-0.001623
80 C	-0.148365	-0.018321
81 C	-0.005481	0.008326
82 H	0.021427	-0.000268
83 C	-0.017587	0.002232
84 H	0.0231	-0.000073
85 C	-0.004008	-0.000431
86 H	0.024159	-0.000012
87 C	-0.010147	0.006795
88 H	0.025259	-0.000282
89 C	0.025696	-0.001137
90 H	0.044083	0.000143
91 C	-0.045728	0.170171
92 C	0.155357	-0.088407
93 C	-0.022493	0.171449
94 C	-0.02645	-0.107368
95 C	0.00739	0.208661
96 C	-0.02652	-0.102963
97 H	0.047253	-0.009704
98 H	0.032153	-0.007855
99 H	0.036484	0.003953

100 H	0.033947	-0.009923
101 H	0.035443	0.003777
102 N	-0.305861	0.381006
103 C	0.049929	-0.026267
104 H	0.065973	0.000513
105 H	0.093365	0.028245
106 H	0.102924	0.024378
107 C	0.047748	0.018053
108 H	0.064122	0.002951
109 H	0.088526	0.013141
110 H	0.074752	0.03045

Supplementary Table 11. A summary of the light yield (LY), limit of detection (LOD) and spatial resolution (SR) of TPP-3C2B:DMA, reported perovskite scintillators and reported organic scintillators.

Scintillator	LY (photons MeV^{-1})	LOD (μGy min^{-1})	SR (lp mm^{-1})	literatures
$CsPbBr_3$ QDs	—	0.78	~10	*Nature* **561**, 88-93 (2018)
$CsPbBr_3$ NCs	177000	—	12.5	*Adv. Mater.* **30**, 1801743 (2018)
Rb_2CuBr_3	91056	7.29	—	*Adv. Mater.* **31**, 1904711 (2019)
Cs_2ZrCl_6-PDMS	49400	3.9	18	*Adv. Mater.* **34**, 2204801 (2022)
$CsPbBr_3$ NCs	9000	—	—	*Nat. Nanotechnol.* **15**, 462-468 (2020)
$CsPbBr_3$ -PPO	—	—	3.5	*Light Sci. Appl.* **9**, 156 (2020)
$CsPbBr_3$:1.5%Eu	10100	—	15	*Adv. Mater.* **33**, 2102529 (2021)
$CsPbBr_3$ NCs	—	2.41	8.0	*Laser Photonics Rev.* **16**, 2100736 (2022)
$Cs_3Cu_2I_5$-PDMS	48800	2.92	17	*ACS Energy Lett.* **7**, 844-846 (2022)
$Cs_5Cu_3Cl_6I_2$	64800-67200	0.66	27.1	*Sci. Adv.* **9**, eadh1789 (2023)
DMAc-TRZ	73500	103.2	16.6	*Nat. Mater.*, **21**, 210-216 (2022)
TPE-Br	10000	200	16.3	*ACS Mater. Lett.*, **4**, 1668-1675 (2022)
o-ITC	—	33	—	*Nat. Photon.*, **15**, 187-192 (2021)
TADF-Br	17619	45.5	18	*Nat. Photon.*, **16**, 869-875 (2022)
CzPADB	—	110	10	Adv. Mater., **36**, 2409338 (2024)
**TPP-3C2B:DMA**	**65535**	**5.98**	**24.6**	**This work**

Supplementary Table 12. Theoretical and experimental values of cell parameters before and after doping.

	TPP-3C2B (Theoretical)	TPP-3C2B (Experimental)	TPP-3C2B:DMA (200:1) (Theoretical)	TPP-3C2B:DMA (200:1) (Experimental)
a (Å)	11.0677	11.0908	11.0600	11.0970
b (Å)	10.2310	10.1179	10.2290	10.1207
c (Å)	17.6082	17.5478	17.5156	17.5511
α (°)	90.0364	90	90.0353	90
β (°)	100.9943	104.846	100.9683	104.823
γ (°)	89.8505	90	89.8662	90
Volume (Å^3)	1946.2359	1903.4	1945.4389	1905.55
